# Supplementary material for: The Roots of Neorautanenia mitis (A. Rich) Verdcourt: Further Evidence of Its Antidiarrhoeal Activity
Source: Molecules. 2023 Jan 9;28(2):673. doi: 10.3390/molecules28020673 (PMC9863173; doi:10.3390/molecules28020673)
Supplement: Supplementary file 1 [file molecules-28-00673-s001.zip › molecules-2091520-supplementary.pdf]

Supplementary Materials

The Roots of *Neorautanenia mitis* (A. Rich) Verdcourt; Further evidence on its anti-diarrheal activity

Christiana J. Dawurung[1,2]*, Joy Gararawa Usman[3], Jurbe G. Gotep[3], and Stephen G. Pyne[1].

[1] School of Chemistry and Molecular Bioscience, Faculty of Science Medicine and Health, University of Wollongong, Wollongong, New South Wales, 2522, Australia.

[2] Department of Veterinary Physiology, Biochemistry and Pharmacology University of Jos, Jos Plateau State, Nigeria.

[3]Drug Development Division, National Veterinary Research Institute (NVRI) Vom, Jos, Plateau State, Nigeria.

*Corresponding author email: golkada@yahoo.com

CONTENTS

^{1}H NMR Spectra……………………………………………….…………………………...4

Figure S1. ^{1}H NMR Spectrum of Compound **1** (500 MHz, $CDCl_3$)………………….........…4

Figure S2. ^{1}H NMR Spectrum of Compound **2** (400 MHz, $CDCl_3$) ……..…………………....5

Figure S3. ^{1}H NMR Spectrum of Compound **3** (400 MHz, $CDCl_3$)……………………….....6

Figure S4. ^{1}H NMR Spectrum of Compound **4** (400 MHz, $CDCl_3$)………………….................7

Figure S5. ^{1}H NMR Spectrum of Compound **6** (400 MHz, $CDCl_3$)….……………...............8

Figure S6. ^{1}H NMR Spectrum of Compound **7** (500 MHz, $CDCl_3$)….…………………………9

Figure S7. ^{1}H NMR Spectrum of Compound **8** (500 MHz, $CDCl_3$)...……………………......10

Figure S8. ^{1}H NMR Spectrum of Compound **9** ((400 MHz, $CDCl_3$)...………………………..11

Figure S9. ^{1}H NMR Spectrum of Compound **10** (400 MHz, $CDCl_3$)..……………….............12

Figure S10. ^{1}H NMR Spectrum of Compound **11** (500 MHz, $CDCl_3$)………………………..13

Figure S11. ^{1}H NMR Spectrum of Compound **12** (500 MHz, $CDCl_3$)……………….……...14

Figure S12. ^{1}H NMR Spectrum of Compound **13** (400 MHz, $CDCl_3$)…….………………..15

Figure S13. ^{1}H NMR Spectrum of Compound **14** (500 MHz, $CDCl_3$)….…………………..16

Figure S14. ^{1}H NMR Spectrum of Compound **15** (400 MHz, $CDCl_3$)…………………….17

Figure S15. ^{1}H NMR Spectrum of Compound **16** (500 MHz, $CDCl_3$)………………........18

Figure S16. ^{1}H NMR Spectrum Compound **17** (500 MHz, $CDCl_3$)…................................19

Figure S17. ^{1}H NMR Spectrum Compound **18** (400 MHz, $CDCl_3$)...........................……..20

Figure S18. ^{1}H NMR Spectrum of Compound **19** (500 MHz, MeOD)…………….........21

Figure S19. ^{1}H NMR Spectrum of Compound **20** (500 MHz, MeOD)……………………...22

Tables……………………………….………………………………………….............23

Table S1. Experimental and Literature^{1}H NMR data of compound 1……………….......23

Table S2. Experimental and Literature ^{1}H NMR data of compound 4……………………...24

Table S3. Experimental and Literature ^{1}H NMR and ^{13}Carbon data of compound 5……...25

Table S4. Experimental and Literature ^{1}H NMR data of compound 12……………………26

Table S5. Experimental and Literature ^{1}H NMR data of compound 13 ……………………27

Table S6. Experimental and Literature ^{1}H NMR data of compounds 11 and 15…………...28

Table S7. Experimental and Literature ^{13}C NMR data of Compounds 19 and 20 ………...30

Table S8. Experimental and Literature ^{13}C NMR of Data of compounds 2, 6, 7, 9, 10…...32

Table S9. Experimental and Literature ^{13}C NMR of Data of compounds 14, 16, 17, 18….33

Table S10. Crystal Data and Structure Refinement for Compound 11……………………34

Table S11. Crystal Data and Structure Refinement for Compound 20……………………35

Table S12. Melting Point and Optical rotation of Compounds 1-20……………………...36

Compound 8 Spectra……………………………………………………………......37

Figure S20. ^{13}CNMR Spectrum of Compound **8** (126 MHz, $CDCl_3$)………………………..37

Figure S21. DEPT Spectrum of Compound **8** (500 MHz, $CDCl_3$)…………………………..38

Figure S22. HSQC Spectrum of Compound **8** (500 MHz, $CDCl_3$)………………………….39

Figure S23. HSQC Spectrum of Compound **8** (500 MHz, $CDCl_3$)………………………….40

Figure S24. COSY Spectrum of Compound **8** (500 MHz, $CDCl_3$)………………………….41

MS for compound 8………………………………………………………………….....42

References……………………………………………………………………………….43

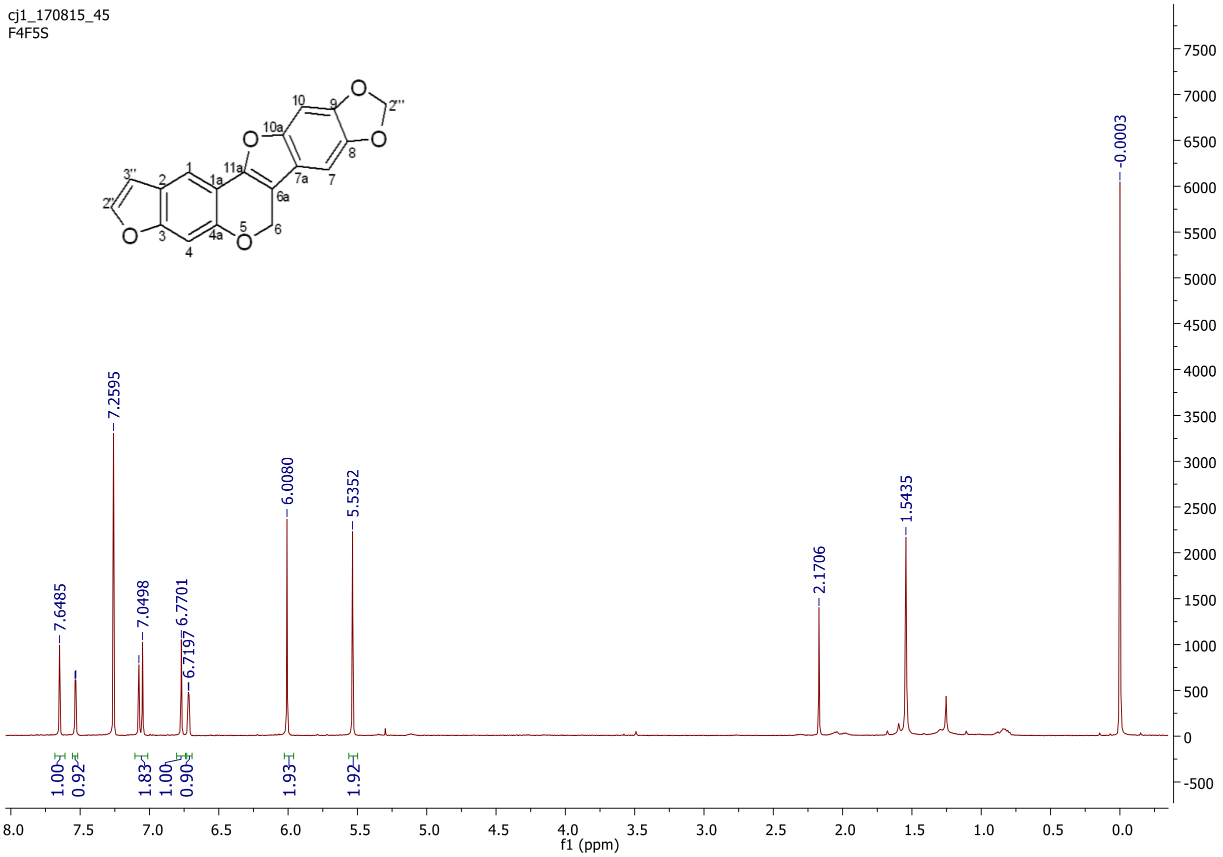

Figure S1. ^{1}H NMR Spectrum of Compound **1** (500 MHz, $CDCl_3$)

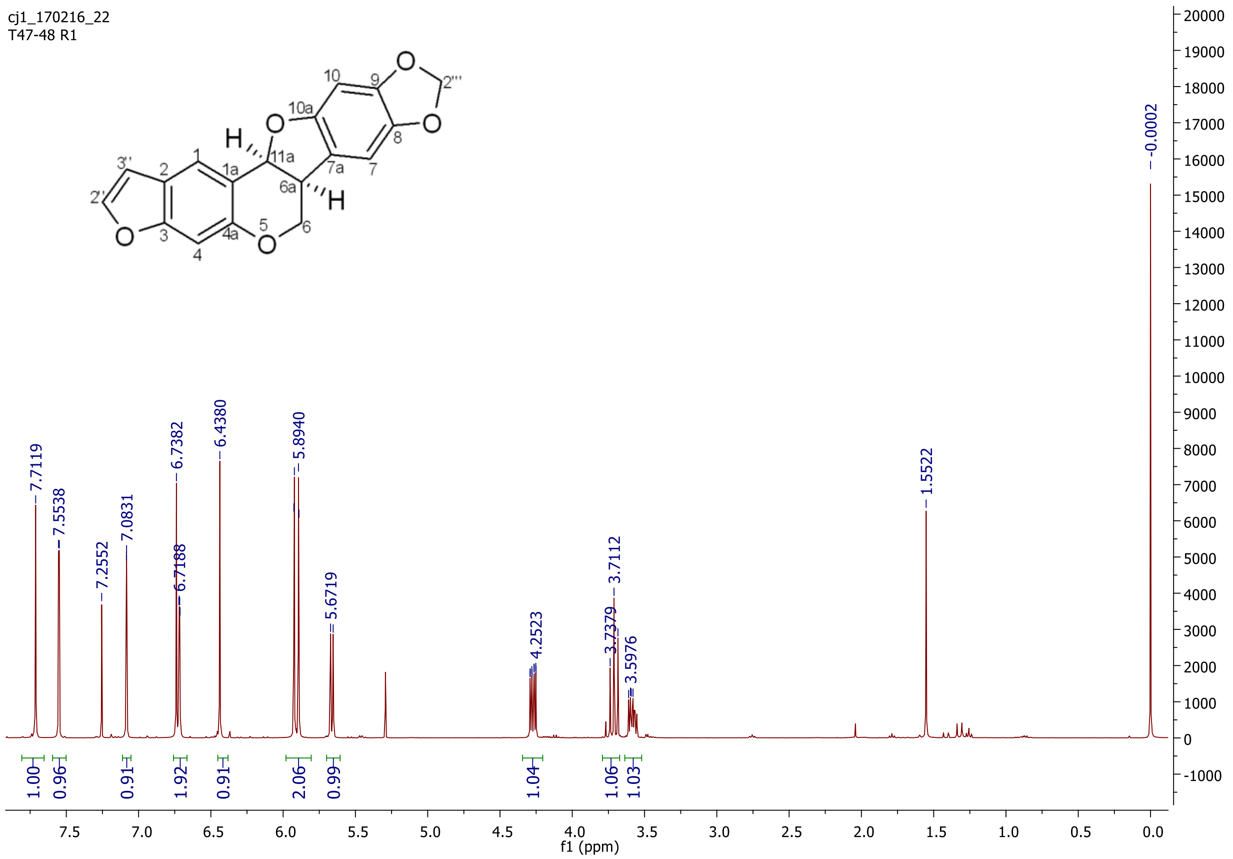

Figure S2. ^{1}H NMR Spectrum of Compound **2** (400 MHz, $CDCl_3$)

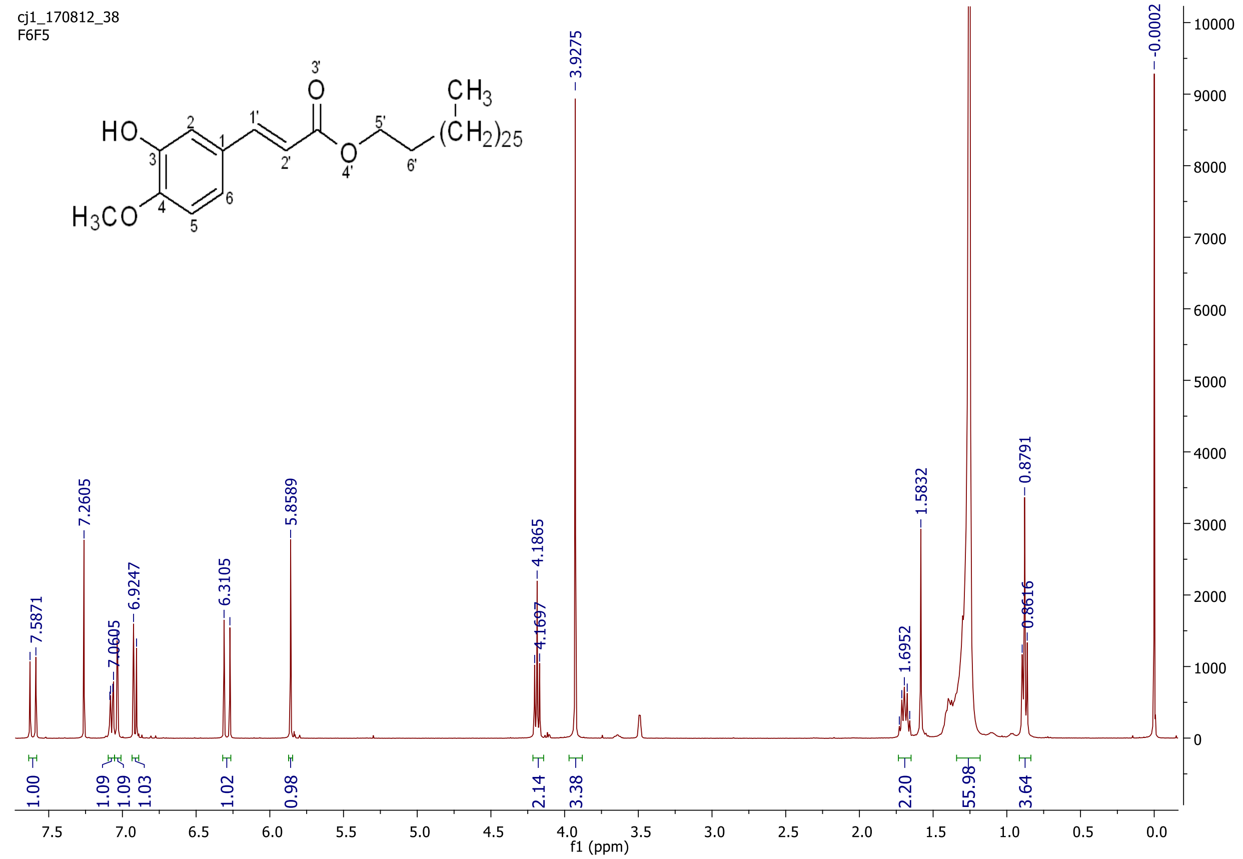

Figure S3. ^{1}H NMR Spectrum of Compound **3** (400 MHz, $CDCl_3$)

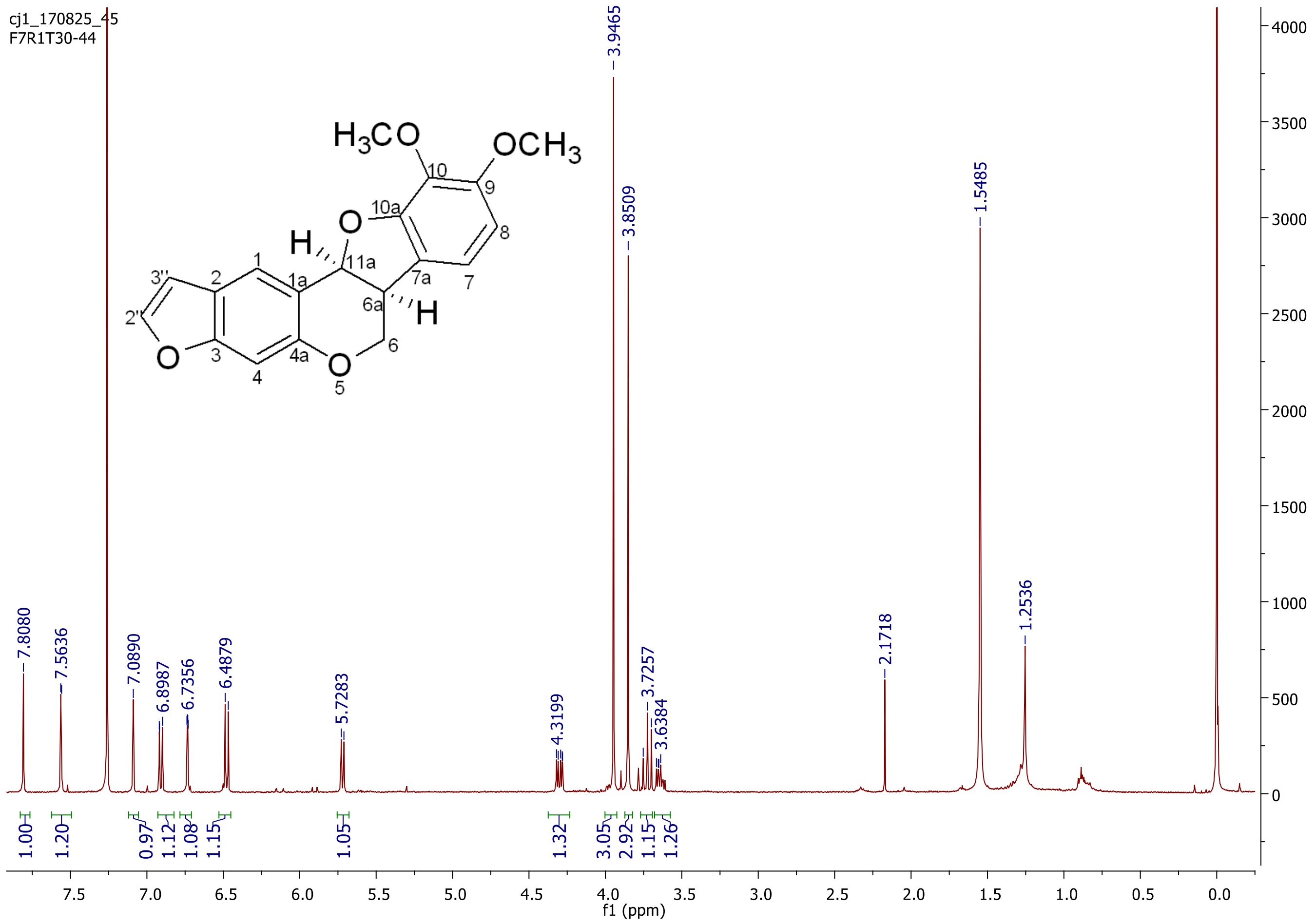

Figure S4. ^{1}H NMR Spectrum of Compound **4** (400 MHz, $CDCl_3$)

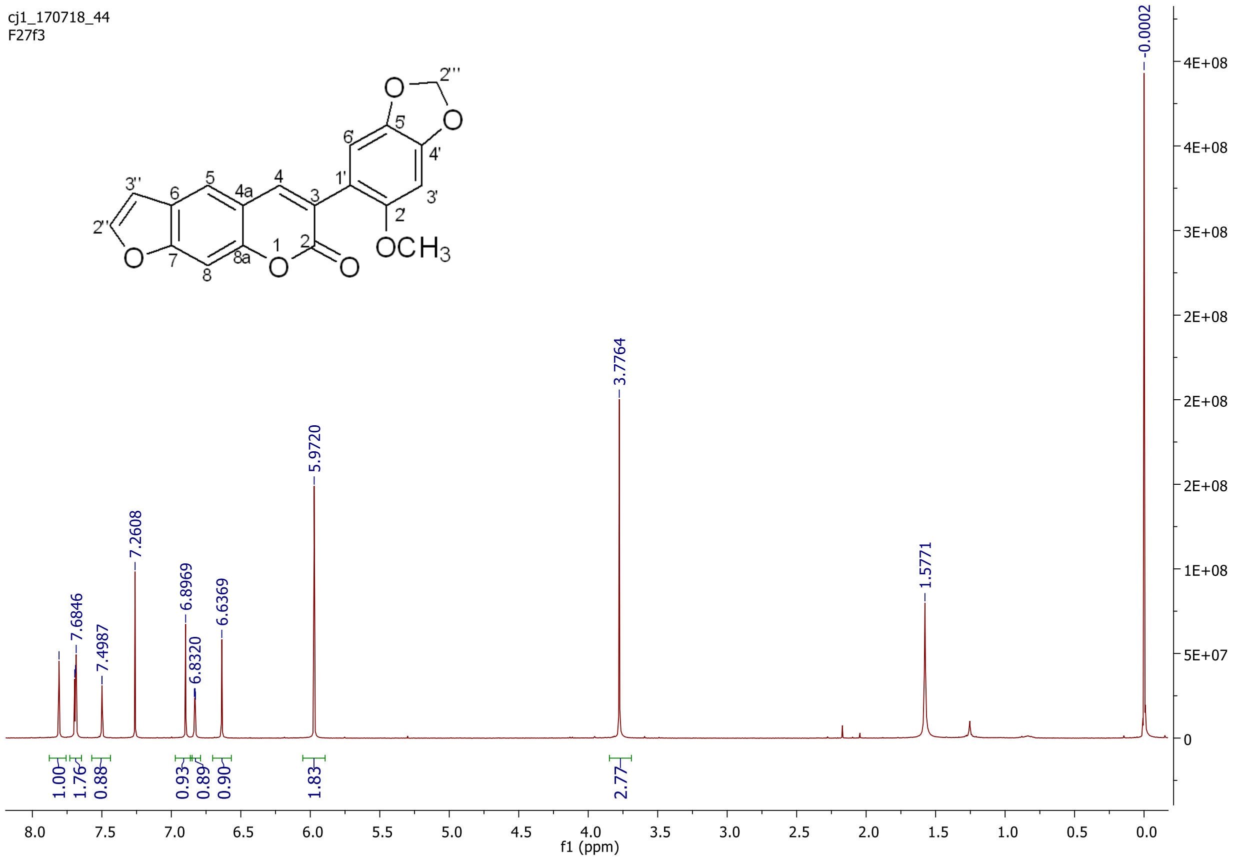

Figure S5. ^{1}H NMR Spectrum of Compound **6** (400 MHz, $CDCl_3$)

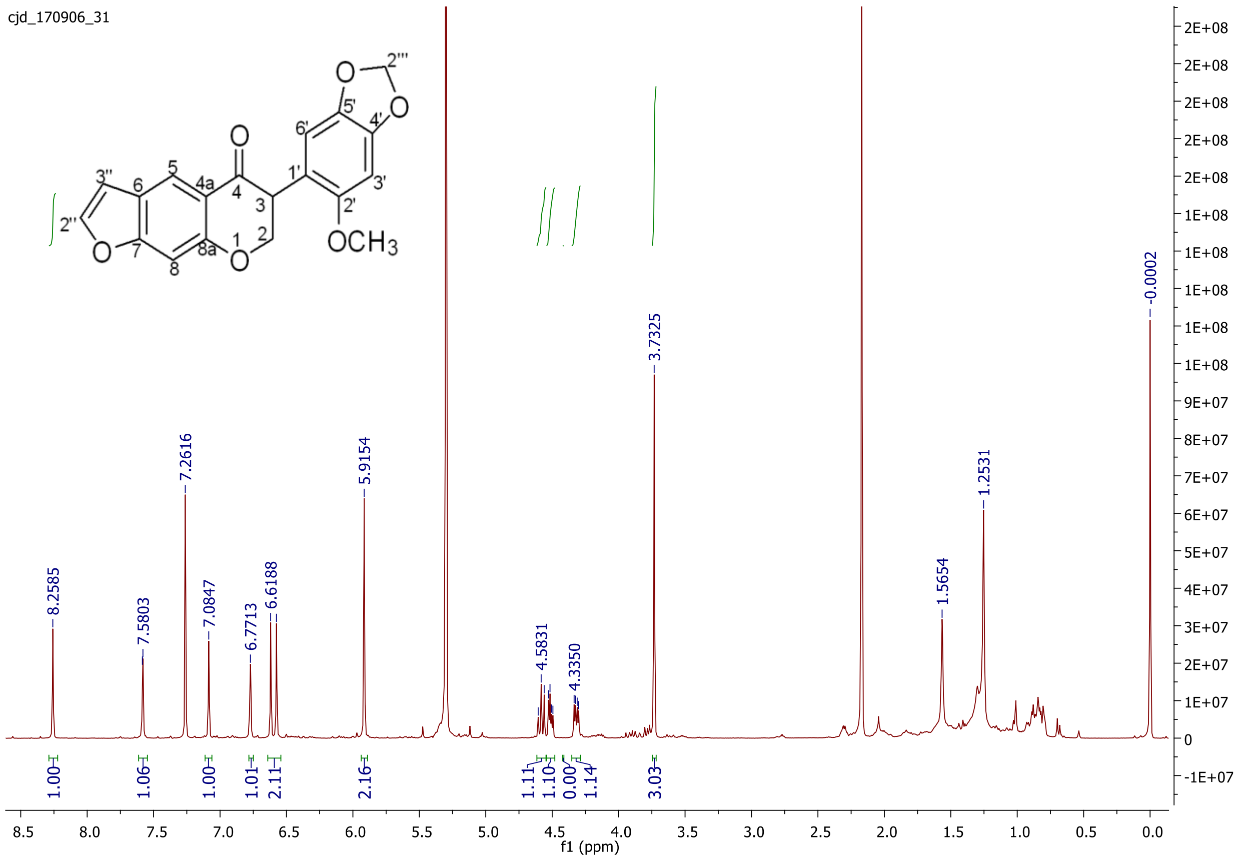

Figure S6. ^{1}H NMR Spectrum of Compound **7** (500 MHz, $CDCl_3$)

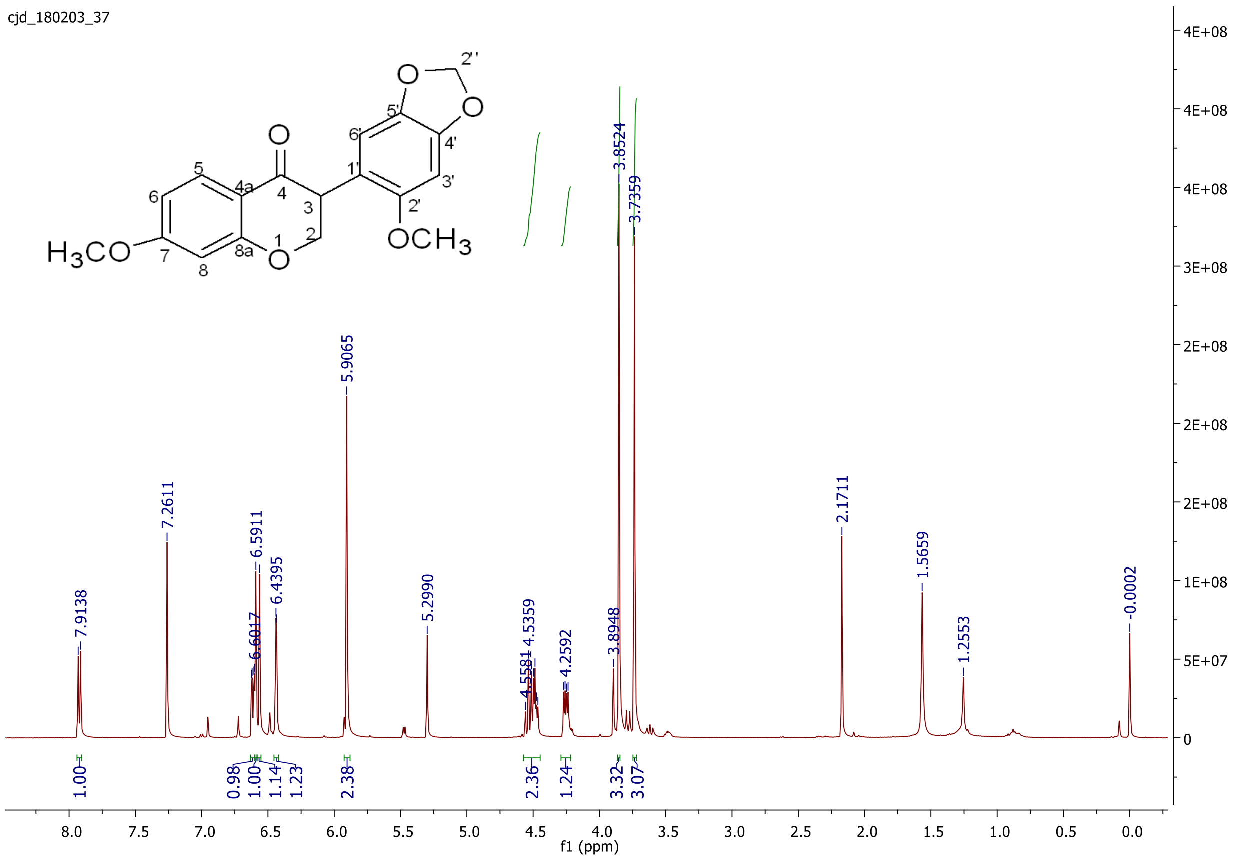

Figure S7. ^{1}H NMR Spectrum of Compound **8** (500 MHz, $CDCl_3$)

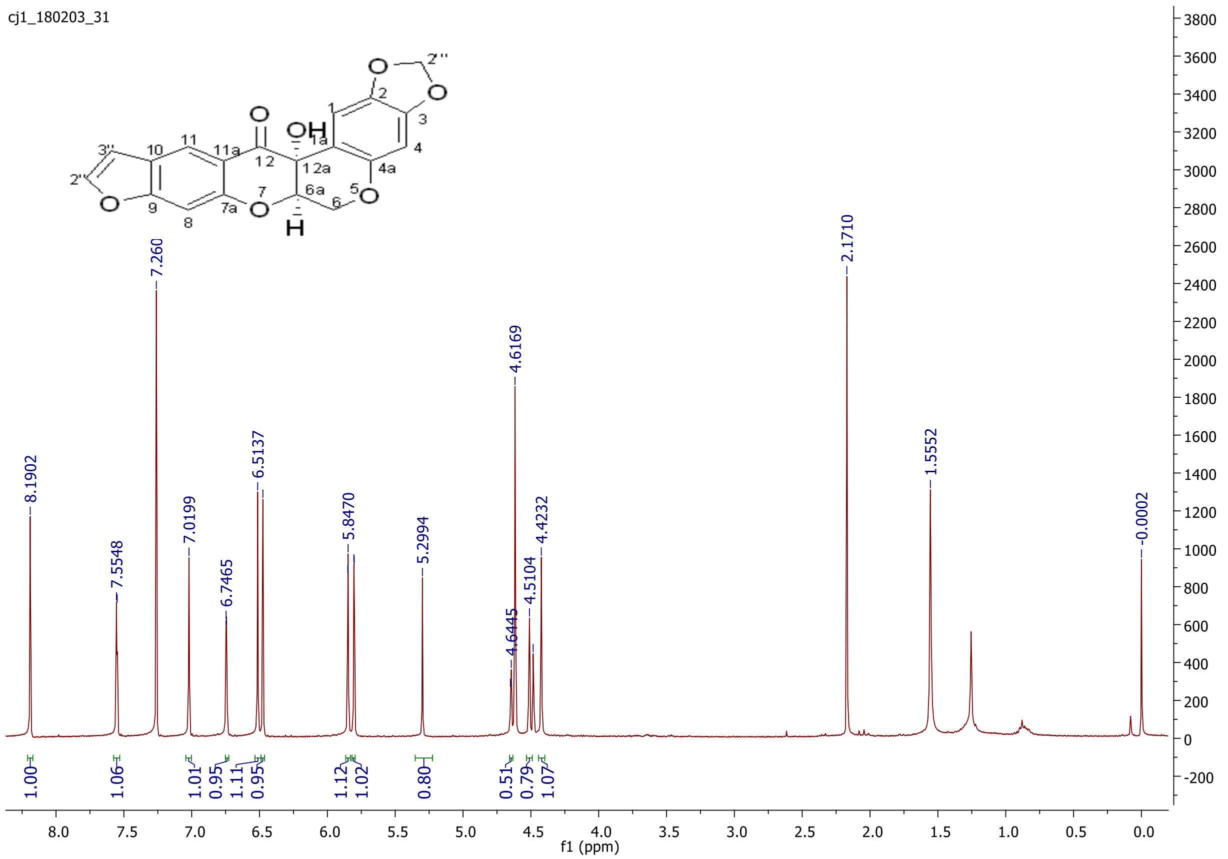

Figure S8. ^{1}H NMR Spectrum of Compound **9** ((400 MHz, $CDCl_3$)

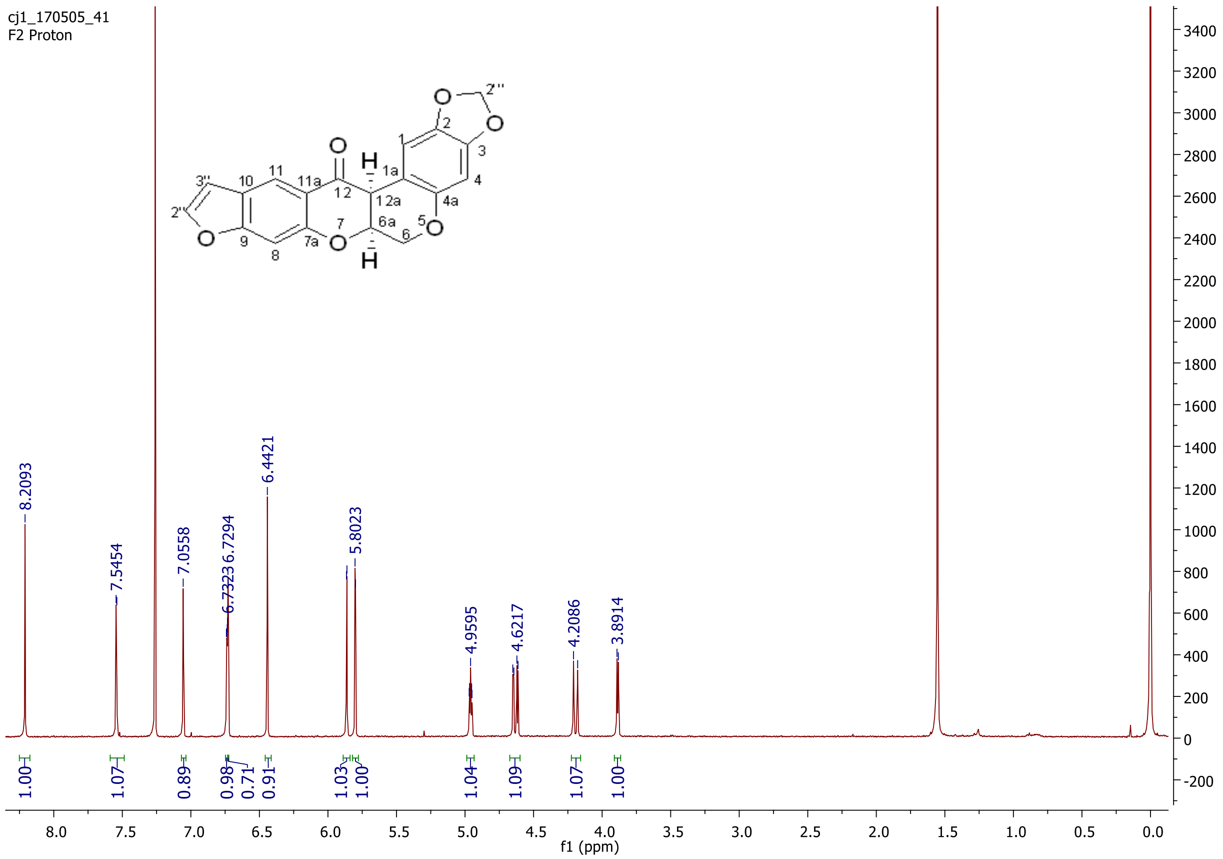

Figure S9. ^{1}H NMR Spectrum of Compound **10** (400 MHz, $CDCl_3$)

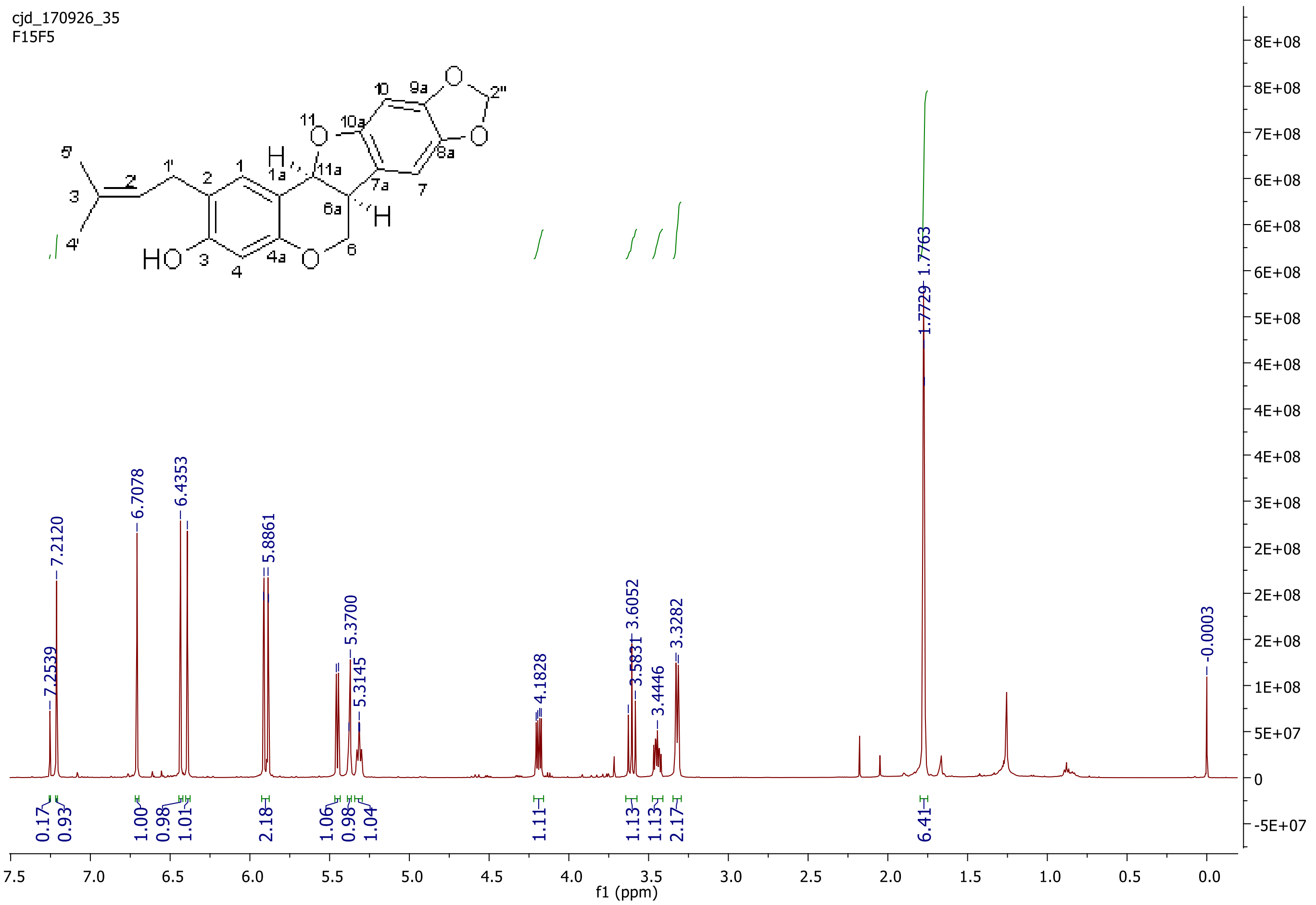

Figure S10.^{1}H NMR Spectrum of Compound **11** (500 MHz, $CDCl_3$)

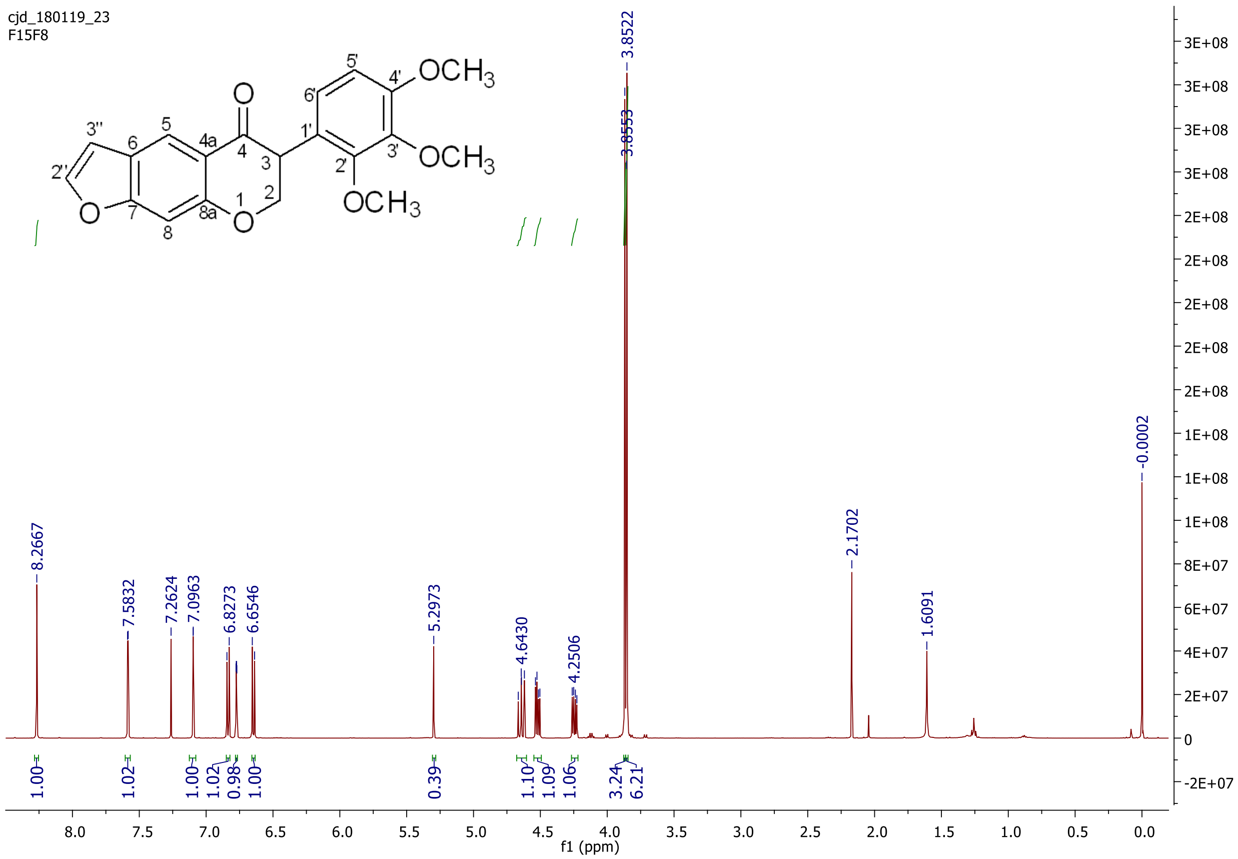

Figure S11. ^{1}H NMR Spectrum of Compound **12** (500 MHz, $CDCl_3$)

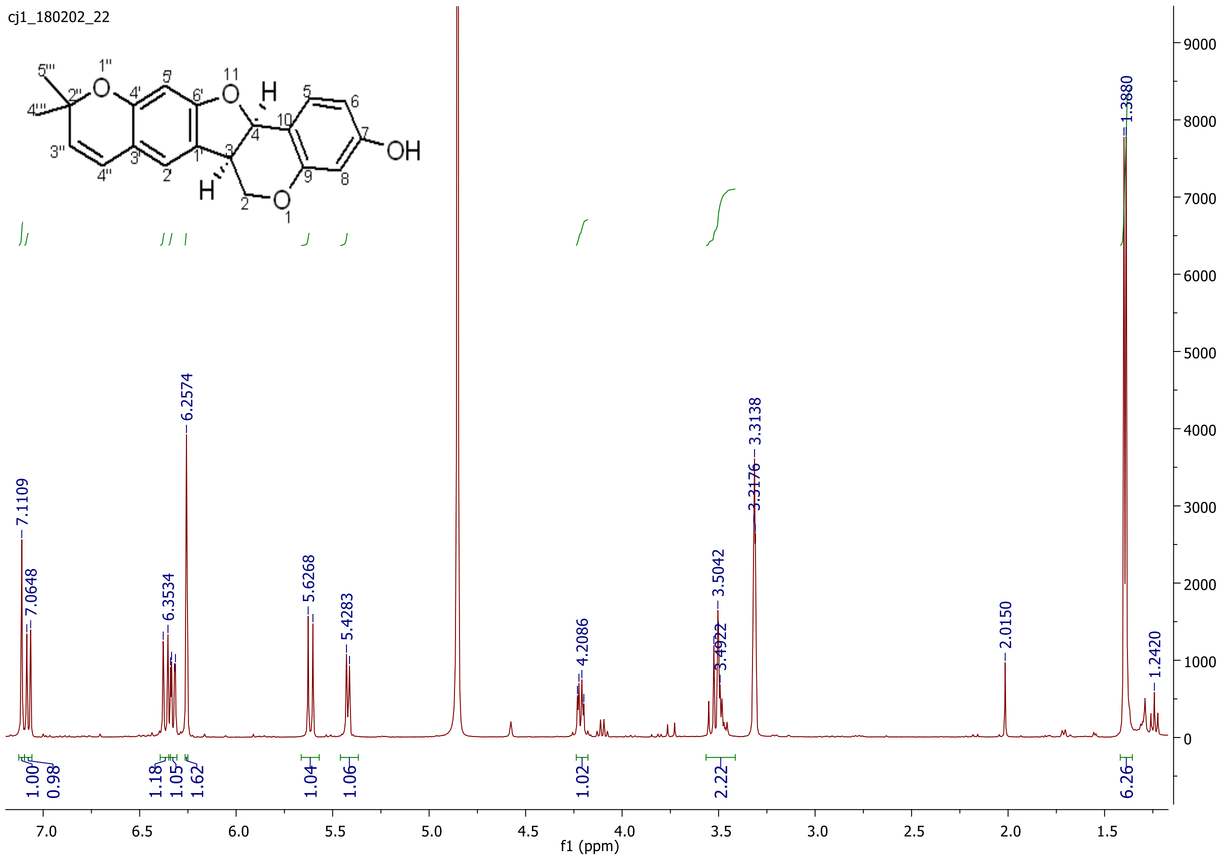

Figure S12. ^{1}H NMR Spectrum of Compound **13** (400 MHz, $CDCl_3$)

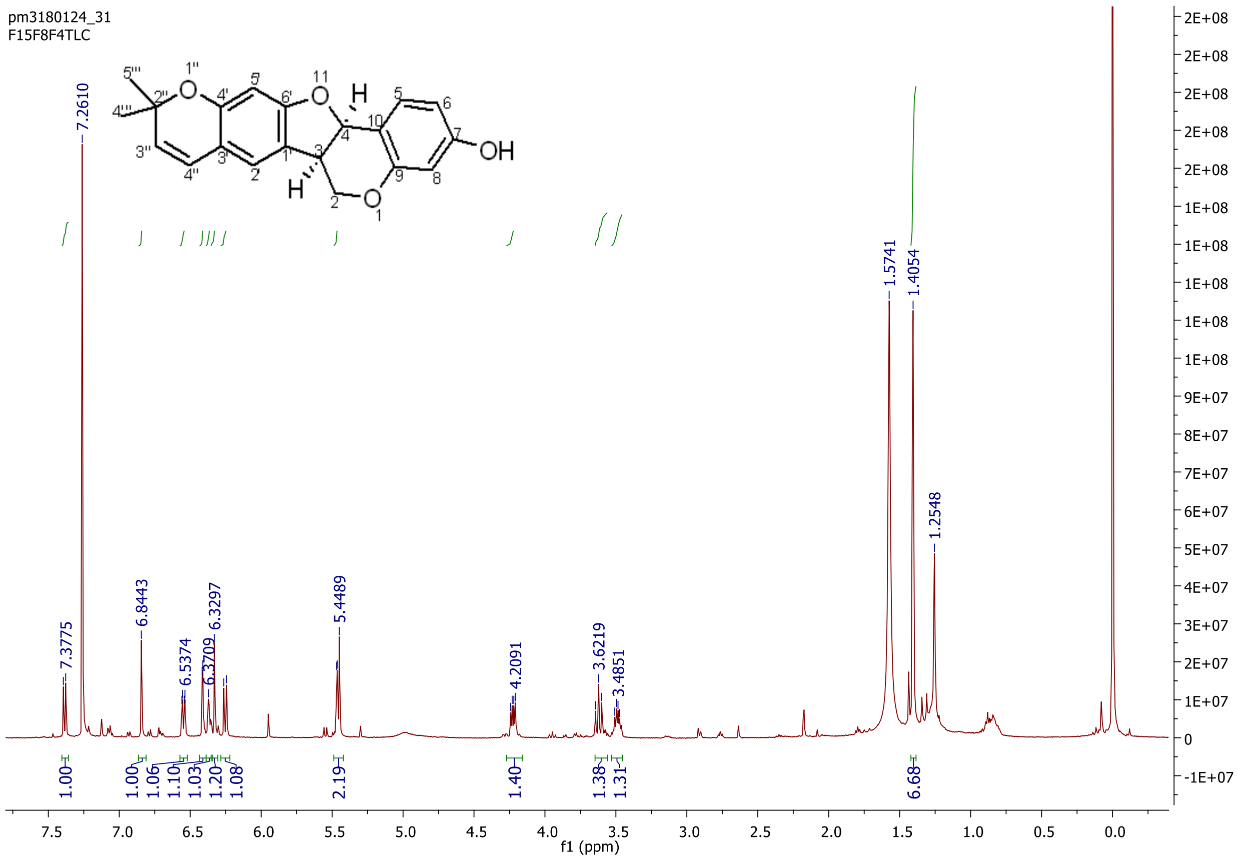

Figure S13. ^{1}H NMR Spectrum of Compound **14** (500 MHz, $CDCl_3$)

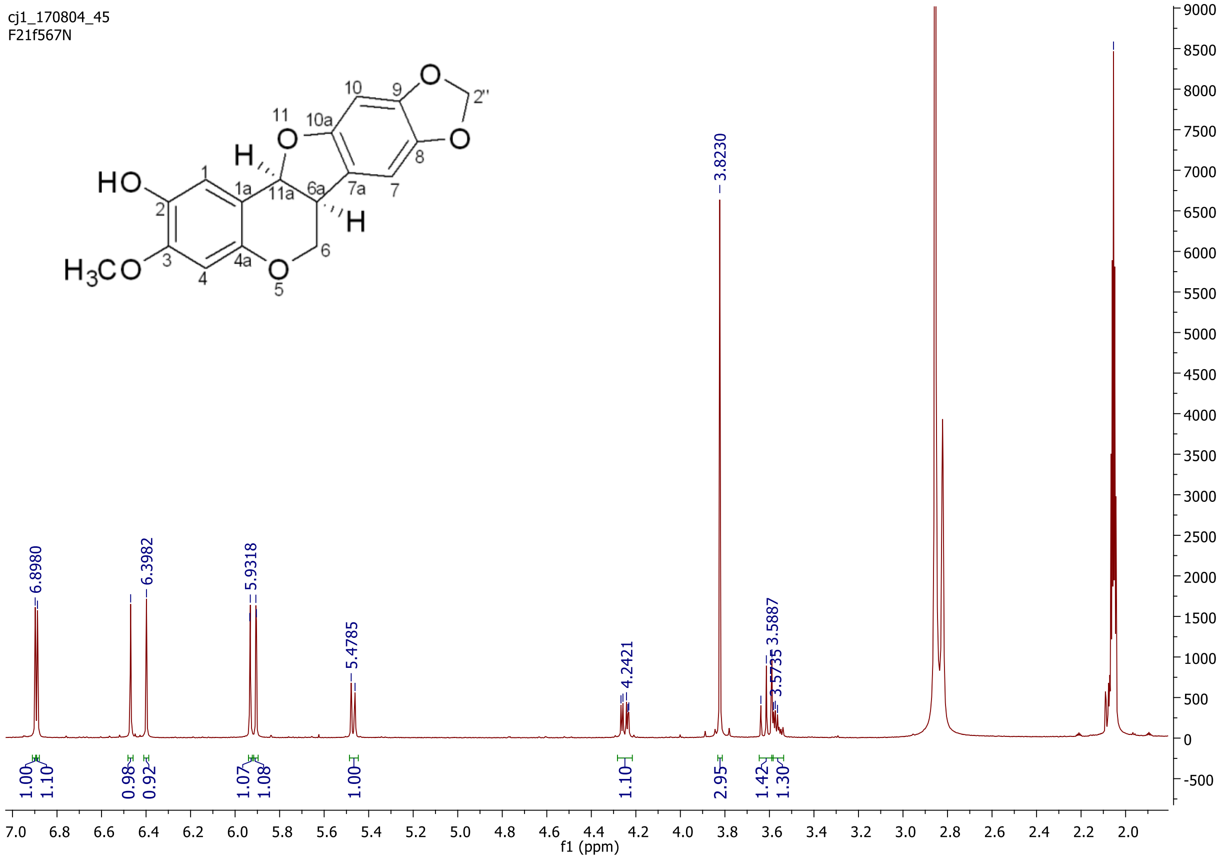

Figure S14. ^{1}H NMR Spectrum of Compound **15** (400 MHz, $CDCl_3$)

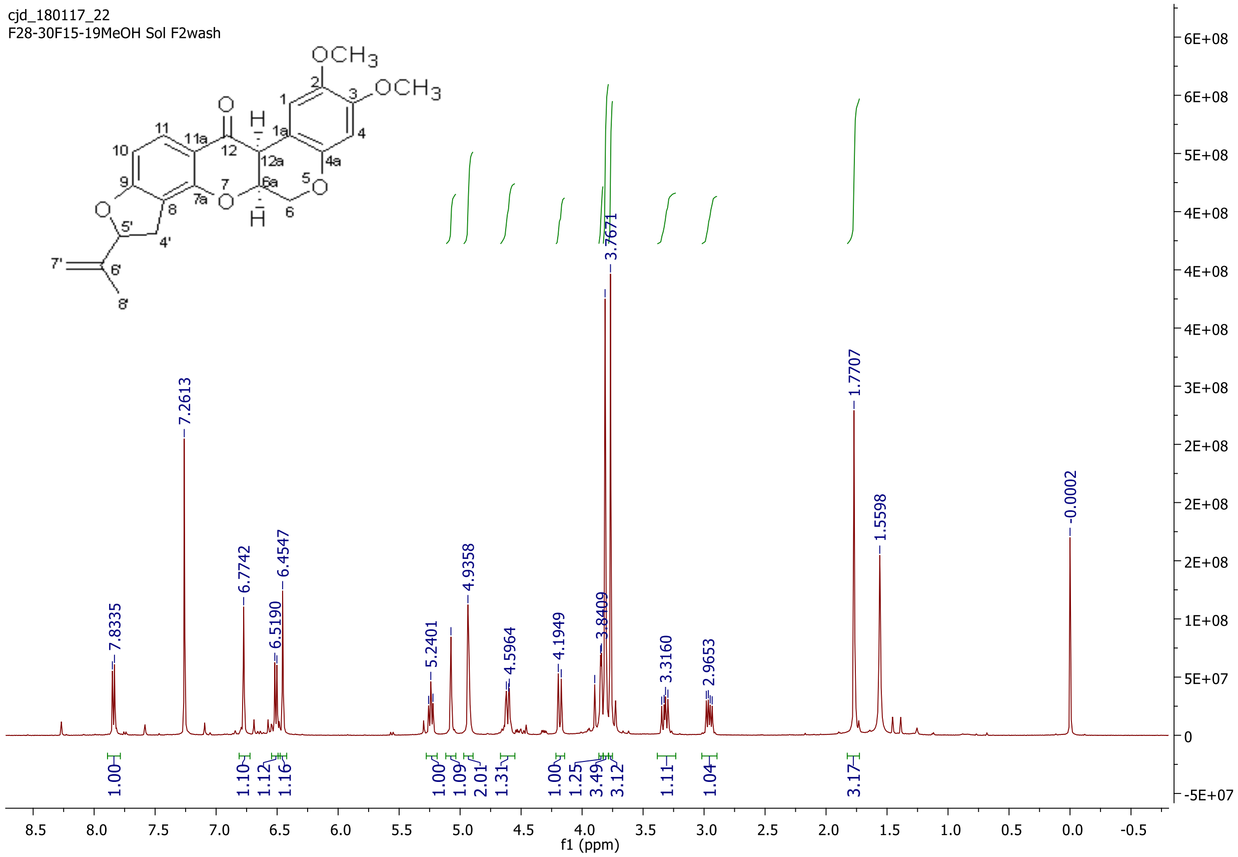

Figure S15. ^{1}H NMR Spectrum of Compound **16** (500 MHz, $CDCl_3$)

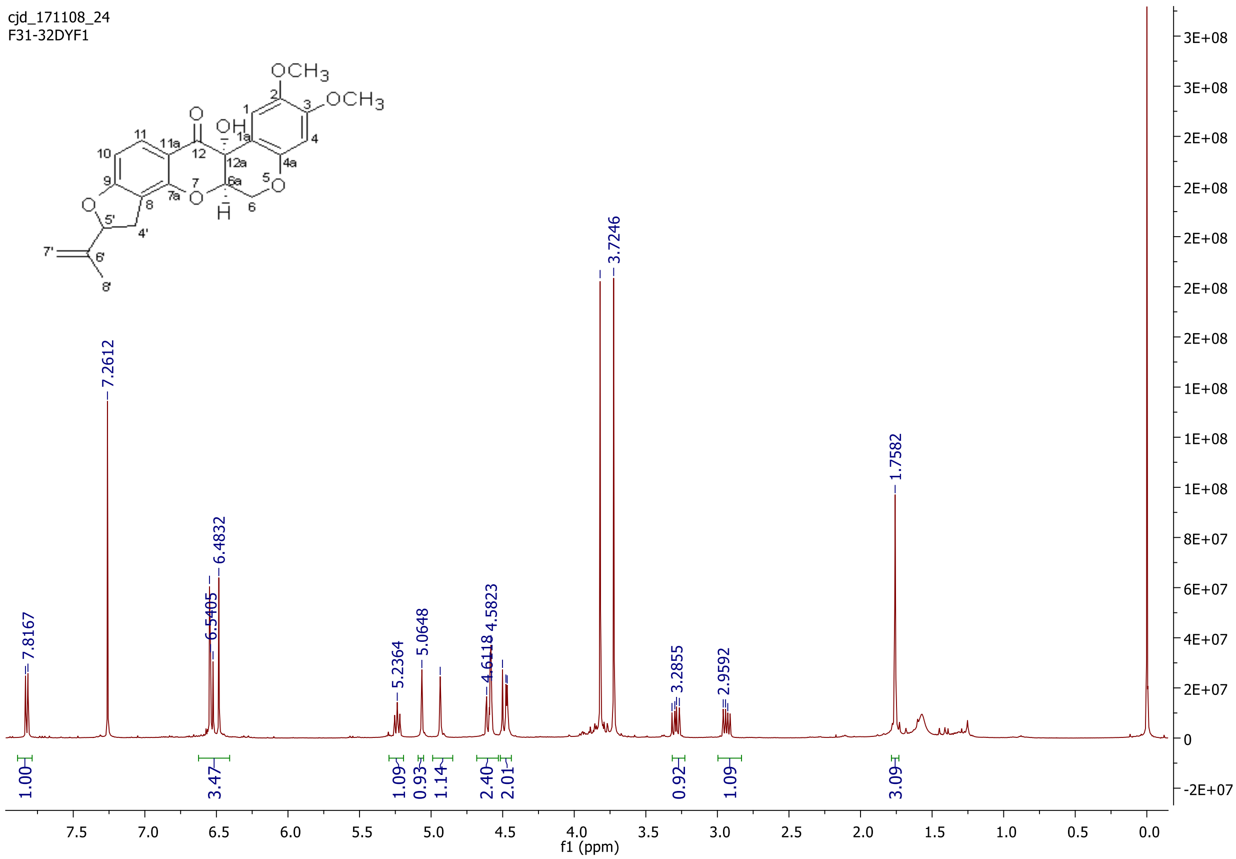

Figure S16. ^{1}H NMR Spectrum Compound **17** (500 MHz, $CDCl_3$)

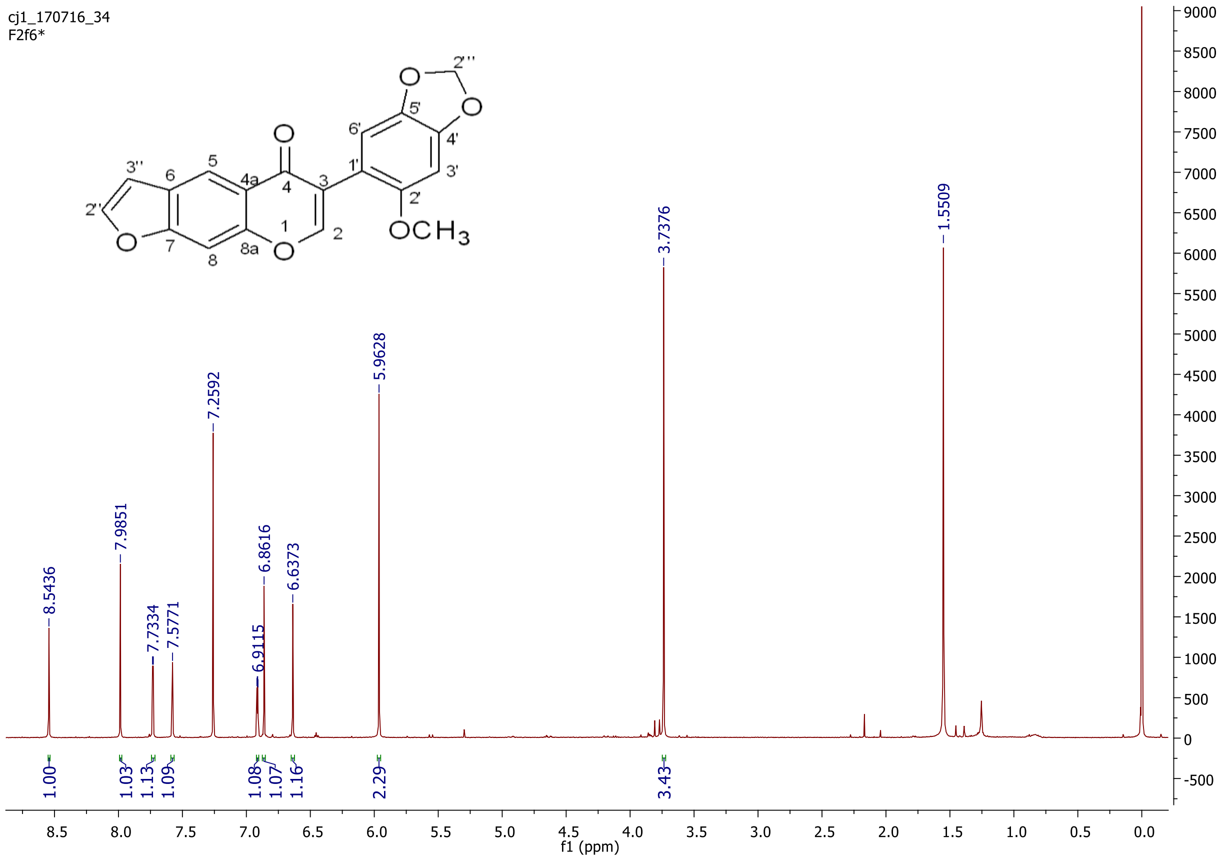

Figure S17. ^{1}H NMR Spectrum Compound **18** (400 MHz, $CDCl_3$)

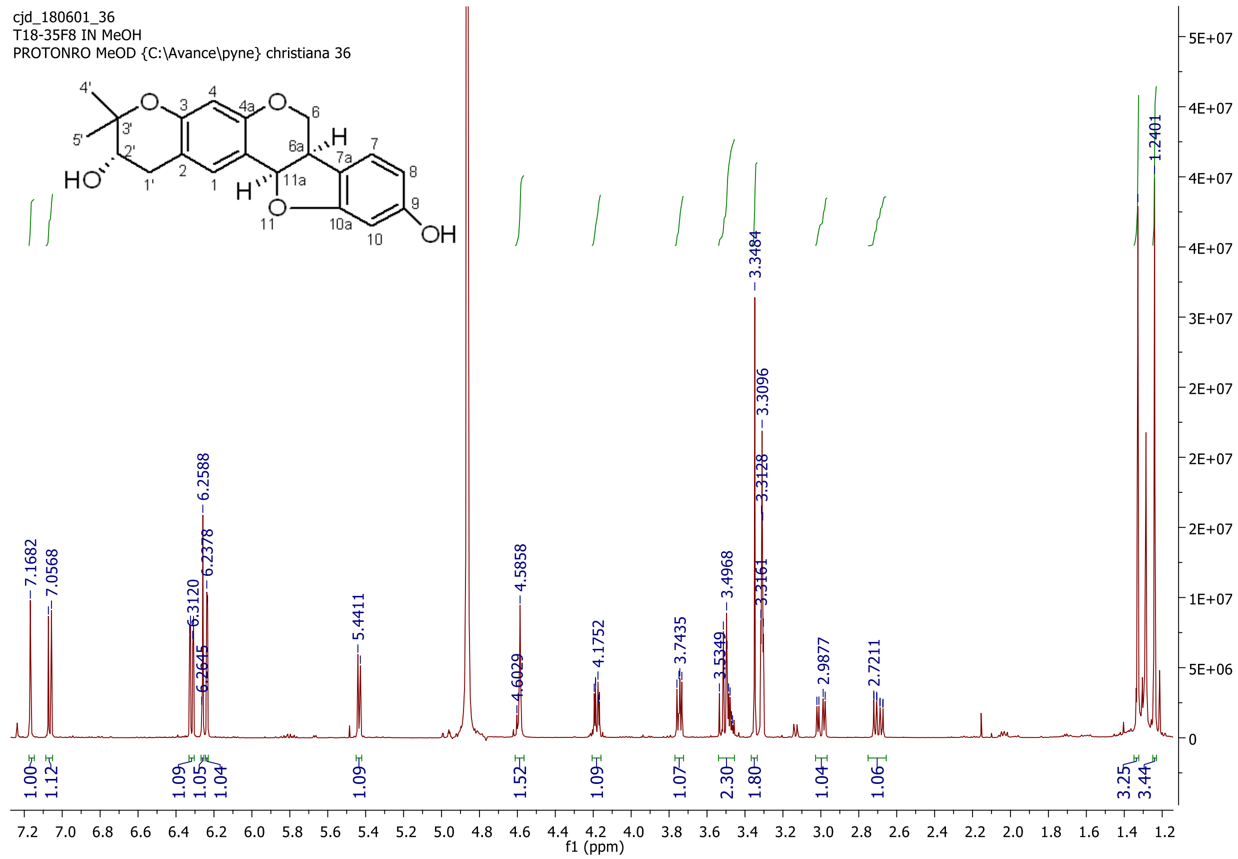

Figure S18. ^{1}H NMR Spectrum of Compound **19** (500 MHz, MeOD)

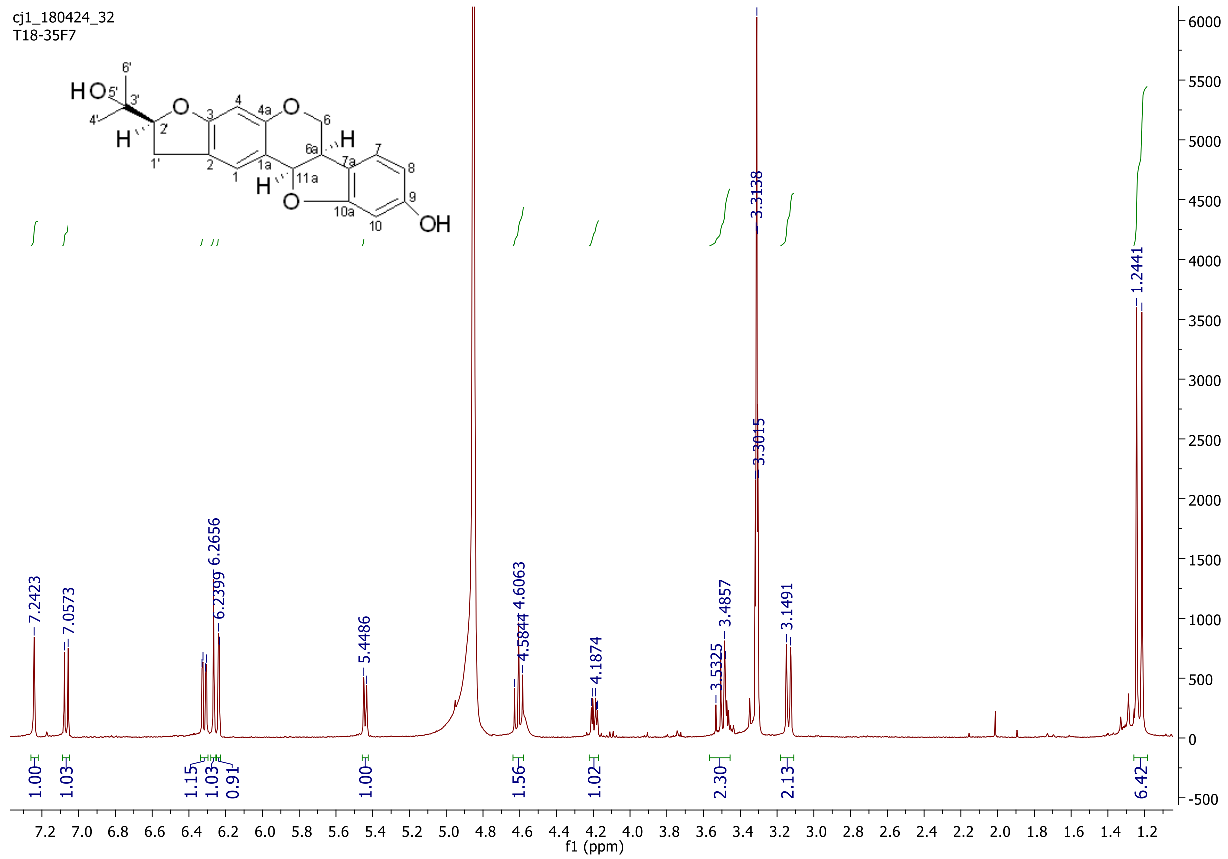

Figure S19. ^{1}H NMR Spectrum of Compound **20** (500 MHz, MeOD)

Table S1. Experimental and Literature ^{1}H NMR Data of Compound **1**

P	H δ (CDCl₃)	
	Expt. (500 MHz)	Lit.[1] (MHz)
1	7.65 (s, 1H)	7.63 (s)
4	7.08 (s, 1H)	7.06 (s)
6	5.54 (s, 2H)	5.51 (s, 2H)
7	6.74 (s, IH)	6.75 (s)
8	--------	--------
10	7.05 (s, 1H)	7.51 (s)
2′′	7.53(d, *J* = 2.1, 1H)	7.56 (d, *J*= 2.2)
3′′	6.73(m, IH)	6.69 (d, *J*=2.2)
2′′′	6.01 (s, 2H)	5.98 (s, 2H)

Assignments are made on the basis of COSY, HSQC, HMBC and NOESY correlations, Chemical shift values are in δ (ppm), and Coupling constants (*J*) are in Hz. P = Position

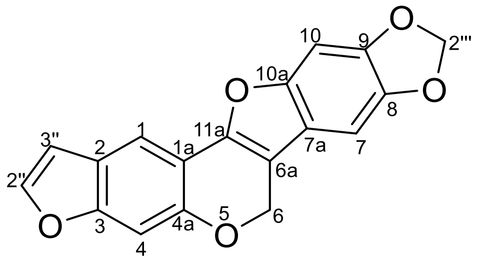

Compound 1 (Neoduleen) Data

$[\alpha]_D^{21}$ +12.9° (*c* 0.3 in $CHCl_3$). ^{1}H NMR (500 MHz, $CDCl_3$) δ7.65 (s, 1H, H-1), 7.53(d, *J* = 2.1, Hz, 1H, H-2′′), 7.08 (s, 1H, H-4), 7.05 (s, 1H, H-10), 6.74 (s, IH, H-7), 6.73(m, IH, H-3′′), 6.01 (s, 2H, H-2′′′), 5.54 (s, 2H, H-6). ^{13}C NMR (126 MHz, $CDCl_3$) δ155.4 (C-3), 151.8 (C-4a), 150.6 (C-10a), 147.9 (C-11a), 145.9 (C-8), 144.8 (C-10), 144.7 (C-2′′), 121.6 (C-2), 119.0 (C-6a), 113.4 (C-1a), 111.9 (C-1), 108.3 (C-7a), 106.6 (C-3′′), 101.5 (C-2′′′), 99.9 (C-4), 97.5 (C-7), 94.1 (C-10), 65.3 (C-6).

Table S2. Experimental and Literature ^{1}H NMR and ^{13}C NMR Data of Compound **3**

**Position**	**Expt. (400 MHz, $CDCl_3$)**		**Lit.[2] (500 MHz)**	
	**^{1}H NMR**	**^{13}C NMR**	**^{1}H NMR**	**^{13}C NMR**
1		127.1		127.1
2	7.03 (d, *J* = 1.9, 1H)	114.7	7.04 (d, *J* = 1.8, 1H)	114.7
3		146.8		146.8
4		147.9		147.9
5	6.91 (d, *J* = 8.1, 1H)	109.3	6.92 (d, *J* = 8.1, 1H)	109.3
6	7.07 (dd, *J* = 8.2, 1.9, 1H)	123.1	7.08 (dd, *J* = 8.1, 1.81H)	123.1
1′	7.61 (d, *J* = 15.9, 1H)	144.6	7.62 (d, *J* = 15.9, 1H)	144.6
2′	6.29 (d, *J* = 15.9, 1H)	115.7	6.29 (d, *J* = 15.9, 1H)	115.7
3′		167.4		167.4
5′	4.19 (t, *J* = 6.7, 2H)	64.6	4.20 (t, *J* = 6.7, 2H)	64.6
6′	1.74 – 1.65 (m, 2H)	29.7	1.70 quint, J=6.8, 2H)	29.7
7′	1.25 (s, 58H, H-7')	29.7-22.7 (-CH_2)$_n$	1.25 (s, 58H, H-7')	29.7-22.7 (-CH_2)$_n$
8′	0.88 (t, *J* = 6.9 Hz, 3H)	14.1 (-CH_3)	0.89 (t, *J* = 6.9, 3H)	14.1 (-CH_3)
OCH_3	3.93 (s, 3H)	55.9	3.94 (s, 3H)	55.9
OH	5.86 (s, 1H)	146.8	5.85 (s. IH)	-----

Assignments are made on the basis of COSY, HSQC, HMBC and NOESY correlations, Chemical shift values are in δ (ppm), and Coupling constants (*J*) are in Hz

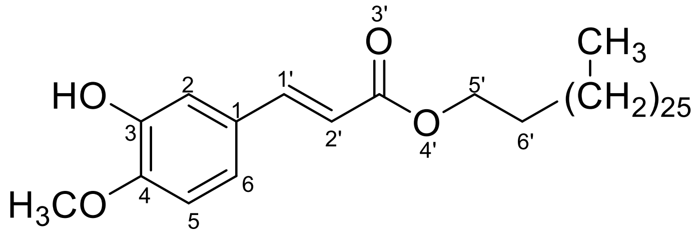

NMR Data of Compound 3 (Ferulic Acid)

Mp 69-71°C and $[\alpha]^{21}$ +18.9° (*c* 0.3 in $CHCl_3$), Its HRESITOFMS data afforded an [M + Na] + ion at *m/z*, implying a molecular formula of $C_{36}H_{62}O_4$ (calcd for $C_{36}H_{62}O_4$, *m/z* 559.4726).

^{1}H NMR (400 MHz, $CDCl_3$) δ 7.61 (d, *J* = 15.9 Hz, 1H, H-1'), 7.07 (dd, *J* = 8.2, 1.9 Hz, 1H, H-6), 7.03 (d, *J* = 1.9 Hz, 1H, H-2), 6.91 (d, *J* = 8.1 Hz, 1H, H-5), 6.29 (d, *J* = 15.9 Hz, 1H, H-2'), 4.19 (t, *J* = 6.7 Hz, 2H, H-5'), 3.93 (s, 3H, OCH_3), 1.74 – 1.65 (m, 2H, H-6'), 1.25 (s, 58H, H-7'), 0.88 (t, *J* = 6.9 Hz, 3H,8').

^{13}C NMR (101 MHz, $CDCl_3$) δ 167.4 (C-3'), 147.9 (C-4), 146.8 (C-3 OH), 144.6 (C-1'), 127.1 (C-1), 123.1 (C-6), 115.7 (C-2'), 114.7 (C-2), 109.3 (C-5), 64.6 (C-5'), 55.9 (OCH_3), 29.7 (C-6'), 29.7-22.7 ($-CH_2)_n$, 14.1 ($-CH_3$).

Table S3. Experimental and Literature ^{1}H NMR Data of Compound **4**

P	H δ (CDCl$_3$)	
	Expt. (400 MHz)	Lit.[3]
1	7.81 (s, 1H)	7.71 (s)
4	7.09 (s, 1H)	7.03 (s)
6	4.33–4.27 (m)	4.22 (m)
	3.73 (t, *J* = 10.7 1H)	3.72 (m)
7	6.91 (dd, *J* = 8.2, 0.6 1H)	6.84 (d)
8	6.48 (d, *J* = 8.2, 1H)	6.41 (d)
10	--------	-------
2′′	7.56(d, *J* = 2.2, 1H)	7.50 (d, *J* = 2.5)
3′′	6.73(dd, *J* = 2.2, 1.0 1H)	6.63 (dd, *J* = 2.5, 1.0)
2′′′	--------	-------
	--------	-------
6a	3.65 (dd, *J* = 6.2, 4.8 1H)	3.53 (m)
11a	5.72 (d, *J* = 6.9 1H)	5.69 (d)
OCH$_3$	3.95 (s, 3H)	3. 91 (s)
	3.85 (s, 3H)	3.81 (s)

Assignments are made on the basis of COSY, HSQC, HMBC and NOESY correlations, Chemical shift values are in δ (ppm), and Coupling constants (*J*) are in Hz. P = Position

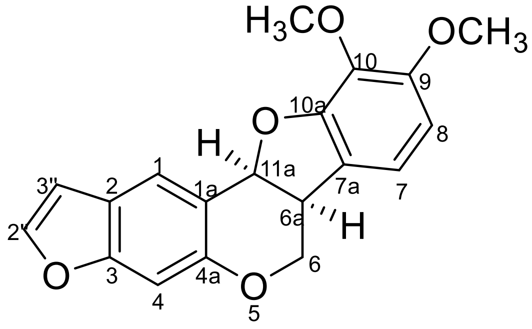

Compound 4 (Ambonane) Data

$[\alpha]^{25}$ -192° (*c* 0.05 in $CHCl_3$) [Lit.[3] $[\alpha]_D$ -214° (*c* 0.01 in $CHCl_3$)], Its HRESITOFMS data afforded an [M+H] + ion at *m/z* 325.1076, implying a molecular formula of $C_{19}H_{16}O_5$ (calcd for $C_{19}H_{16}O_5$, *m/z* 325.1076). ^1H NMR (400 MHz, $CDCl_3$) δ 7.81 (s, 1H, H-1), 7.56 (d, *J* = 2.2 Hz, 1H, H-2"), 7.09 (s, 1H, H-4), 6.91 (dd, *J* = 8.1, 0.6 Hz, 1H, H-7), 6.73 (dd, *J* = 2.2, 1.0 Hz, 1H, H-3"), 6.48 (d, *J* = 8.2 Hz, 1H, H-8), 5.72 (d, *J* = 6.9 Hz, 1H, H-11a), 4.33 – 4.27 (m, 1H, H-6), 3.95 (s, 3H, OCH_3-9), 3.85 (s, 3H, OCH_3-10), 3.73 (t, *J* = 10.7 Hz, 1H, H-6), 3.65 (dd, *J* = 6.2, 4.8 Hz, 1H, H-6a).^{13}C NMR (126 MHz, $CDCl_3$) δ 155.7 (C-3), 153.5 (C-4a), 153.1 (C-9), 151.2 (C-10), 145.1 (2"), 145. (C-10a), 123.3 (C-7a), 122.3 (C-1), 121.7 (C-2), 118.1 (C-7), 116.4 (C-1a), 106.3 (C-3"), 104.8 (C-8), 99.7 (C-4), 79.7 (C-11a), 77.27, 77.01, 76.76, 66.9 (C-6), 60.7 (OCH_3), 56.4 (OCH_3), 40.1 (C-6a).

Table S4. Experimental and Literature ^1H NMR Data of Compound **12**

**P**	**H δ (CDCl₃)**	
	Expt. (400MHz)	Lit.[4]
2	4.64 (dd, *J*= 11.9, 11, 1H)	4.50 (dd)
	4.52 (dd, *J*= 11.0, 5.5, 1H)	4.50 (dd)
3	4.24 (dd, *J*=11.9,5.5, 1H)	4.50 (dd)
5	8.27 (s, 1H)	8.23 (s)
8	7.06 (s, 1H)	7.07 (s)
3′	--------	--------
5′	6.84 (d, *J* = 8.5, 1H)	6.83 (d)
6′	6.65 (d, *J* = 8.5, 1H)	6.75 (d)
2′′	7.59 (d, *J*=2.3, 1H)	7.56 (d)
3′′	6.77 (dd, *J* = 2.3, 1.0 1H)	6.70 (d)
2′′′	--------	--------
OCH_3	3.87 (s, 3H)	3.90 (s)
	3.86 (s, 3H)	3.88 (s)
	3.85 (s, 3H)	3.87 (s)

made

are in

(*J*)

Assignments are on the basis of COSY, HSQC, HMBC and NOESY correlations, Chemical shift values δ (ppm), and Coupling constants are in Hz

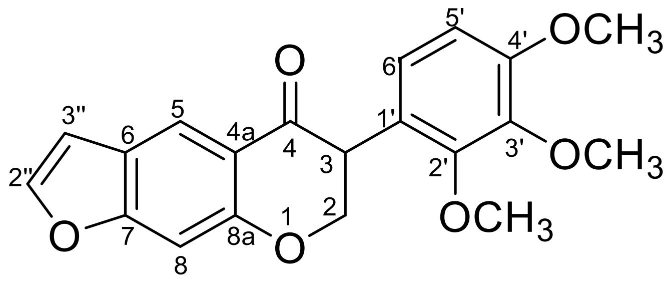

Compound 12 (Nepseudin) Data

[α] [24] -102.2° (*c* 0.8 in $CHCl_3$), [Lit.[4] [α] [20] 0° ($CHCL_3$)] Its HRESITOFMS data afforded an [M + H] + ion at *m/z* 355.1182, implying a molecular formula of $C_{20}H_{18}O_6$ (calcd for $C_{18}H_{17}O_6$, *m/z* 355.1182). ^{1}H NMR (500 MHz, $CDCl_3$) δ 8.27 (s, 1H, H-5), 7.59 (d, *J* = 2.3 Hz, 1H, H-2"), 7.06 (s, 1H, H-8), 6.84 (d, *J* = 8.5 Hz, 1H, H-5'), 6.77 (dd, *J* = 2.3, 1.0 Hz, 1H, H-3"), 6.65 (d, *J* = 8.5 Hz, 1H, H-6'), 4.64 (dd, *J* = 11.8, 11.1 Hz, 1H, H-2), 4.52 (dd, *J* = 11.0, 5.5 Hz, 1H, H-2), 4.24 (dd, *J* = 11.9, 5.5 Hz, 1H, H-3), 3.87 (s, 3H, OCH_3), 3.86 (s, 3H, OCH_3), 3.85 (s, 3H, OCH_3). ^{13}C NMR (101 MHz, $CDCl_3$) δ 192.9 (C-4), 159.9 (C-8a), 159.3 (C-7), 153.7 (C-3'), 152.0 (C-2'), 146.0 (C2"), 142.3 (C-4'), 124.5 (C-5'), 122.7 (C-6), 121.5 (C-1'), 120.9 (C-5), 118.8 (C-4a), 107.4 (C-6'), 107.1 (C-3"), 99.7 (C-8), 71.6 (C-2), 60.7 (OCH_3), 60.7 (OCH_3), 56.0 (OCH_3), 49.0 (C-3).

Table S5. Experimental and Literature ^{1}H NMR Data of Compound **13**

**P**	**H** δ **(CD_3OD)**

	Expt. (400MHz)	Lit. [5, 6]
1	7.11 (s, 1H)	7.16(s)
2	--------	-------
	--------	-------
3	--------	-------
4	6.26 (s, 1H)	6.42 (s)
5	--------	-------
6	4.22 (dd, J = 9.7, 3.6, 1H)	4.22 (m)
	3.51 (dd, J = 7.2, 5.7, 1H)	3.62 (m)
7	7.07 (d, J = 8.0, 1H)	7.06 (d)
8	6.33 (dd, J = 8.0, 2.1, 1H)	6.36 (d)
10	6.29 (d, J = 2.1, 1H)	6.40 (d)
11a	5.42 (d, J =6.3, 1H)	5.49 (d)
2′	----------	---------
3′	5.61 (d, J = 9.8, 1H)	5.56 (d)
4′	6.37 (d, J = 9.8, 1H)	6.33 (d)
5′	--------	---------
2′′	1.39 (s, 3H)	1.41 (s, 3H)
3′′	1.40 (s, 3H)	1.43 (s, 3H)
4′′		
6a	3.36 – 3.27 (m, 1H)	3.52(m)
4′′′	--------	-------
5′′′	--------	-------
OH	2.78 (s)	-------

Assignments are made on the basis of COSY, HSQC, HMBC and NOESY correlations, Chemical shift values are in δ (ppm), and Coupling constants (J) are in Hz. P = Position

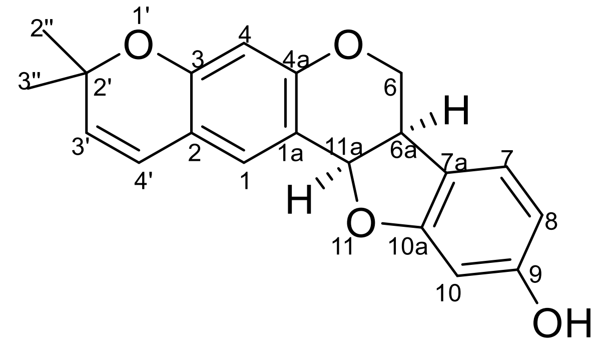

Compound 13 (Neorautenol) Data

Mp 172-174 ˚C, (Lit.[1, 5] 93-95 ˚C) $[\alpha]^{21}$ -127.2˚ (*c* 0.2 in $CHCl_3$) [Lit.[1,5] $[\alpha]_D$ -273˚ (*c* 0.08 in $CHCl_3$)], Its HRESITOFMS data afforded an [M + H] + ion at *m/z* 323.1283, implying a molecular formula of $C_{20}H_{18}O_4$ (calcd for $C_{20}H_{18}O_4$, *m/z* 323.1283). ^{1}H NMR (400 MHz, MeOD) δ 7.11 (s, 1H, H-1), 7.07 (d, *J* = 8.0 Hz, 1H, H-7), 6.37 (d, *J* = 9.8 Hz, 1H, H-4'), 6.33 (dd, *J* = 8.0, 2.1 Hz, 1H, H-8), 6.29 (d, *J* = 2.1 Hz, 1H, H-10) 6.26 (s, 1H, H-4), 5.61 (d, *J* = 9.8 Hz, 1H, H-3'), 5.42 (d, *J* = 6.3 Hz, 1H, H-11a), 4.22 (dd, *J* = 9.7, 3.6 Hz, 1H, H-6), 3.51 (dd, *J* = 7.2, 5.7 Hz, 1H, H-6), 3.36 – 3.27 (m, 1H, H-6a), 1.40 (s, 3H, H-3''), 1.39 (s, 3H, H-2''). ^{13}C NMR (101 MHz, MeOD) δ 161.9 (C-10a), 159.8 (C-9), 157.8 (C-3), 155.7 (C-4a), 130.2 (C-3'), 129.8 (C-1), 125.9 (C-7), 122.7 (C-4'), 119.4 (C-7a), 117.5 (C-1a), 114.2 (C-2), 108.7 (C-8), 105.3 (C-4), 98.8 (C-10), 79.7 (C-2'), 77.6 (C-11a), 67.7 (C-6), 40.9 (C-6a), 28.4 (3''), 28.2 (2'').

Table S6. Experimental and Literature ^{1}H NMR Data of Compounds **11** and **15**

**P**	**H δ 11 ($CDCl_3$)**		**H δ 15 (Acetone)**	
	Expt. (500 MHz)	Lit.[7]	Expt. (400 MHz)	Lit.[8]
1	7.21 (s, 1H)	7.25(s)	6.89 (s, 1H)	7.01 (s)
4	6.44 (s, 1H)	6.47(s)	6.40 (s, 1H)	6.44 (s)
6	4.19 (dd, = 11.0, 5.0, 1H)	4.18	4.27 – 4.23 (m)	4.22 (m)
	3.61 (t, *J* = 11.0, 1H)	3.62	3.65 – 3.58 (m)	3.65 (m)
7	6.71 (s, 1H)	6.73(s)	6.90 (s, 1H)	6.70 (s)
10	6.39 (s, 1H)	6.42(s)	6.47 (s, 1H)	6.42 (s)
1′	3.32 (d, *J* = 7.2, 2H)	3.27-3.38 (d)	--------	--------
2′	5.34 – 5.29 (m, 1H)	5.45-5.22 (m)	--------	--------
4′	1.81 (S, 3H)	1.77(s)	--------	--------
5′	1.76 (S,3H)	1.71(s)	--------	--------
2′′	5.88, 5.91, (d, *J* = 1.4, 2H)	5.92	5.92 (d, *J* = 1.0, 2H)	5.90 (d, 2H)
6a	3.44 (s, 1H)	3.47(s)	3.71 – 3.47 (m, 1H)	3. 45 (m)
11a	5.45 (d, *J* = 6.9, 1H)	5.46	5.47 (d, *J* = 6.9, 1H)	5.43
OCH_3	-------	--------	3.82 (s, 3H)	3.87(s)
OH	5.37 (d, *J* = 4.4, 1H)	5.45	5.62 (s)	5.28 (s)

Assignments are made on the basis of COSY, HSQC, HMBC and NOESY correlations, Chemical shift values are in δ (ppm), and Coupling constants (*J*) are in Hz. P = Position

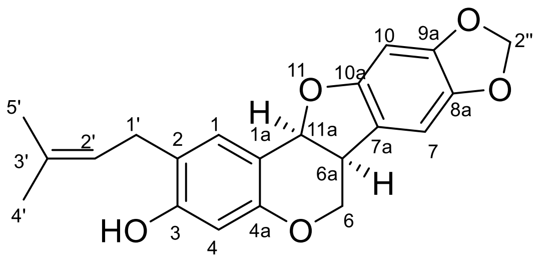

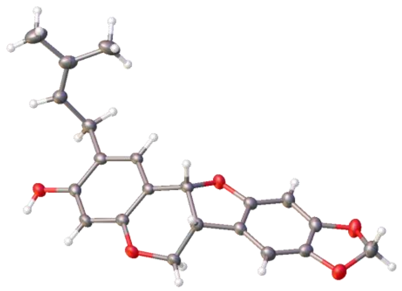

NMR Data of Compound 11 ((-)-2-isopentenyl-3-hydroxy-8-9-methylenedioxypterocarpan)

Mp 149-152°C (Lit.[7] 146-147°C), $[\alpha]^{23}$ -339.5° (*c* 0.1 in $CHCl_3$) [Lit.[7] $[\alpha]^{23}$ -261.9° (*c* 0.8 in $CHCl_3$)], Its HRESITOFMS data afforded an [M-H] + ion at *m/z* 353.2661, implying a molecular formula of $C_{21}H_{20}O_5$ (calcd for $C_{18}H_{17}O_6$, *m/z* 353.2661). ^{1}H NMR (500 MHz, $CDCl_3$) δ 7.21 (s, 1H, H-1), 6.71 (s, 1H, H-7), 6.44 (s, 1H, H-4), 6.39 (s, 1H, H-10), 5.91 (d, *J* = 1.4 Hz, 1H, H-2"), 5.88 (d, *J* = 1.4 Hz, 1H, H-2"), 5.45 (d, *J* = 6.9 Hz, 1H, H-11a), 5.37 (d, *J* = 4.4 Hz, 1H, OH), 5.34 – 5.29 (m, 1H, H-2'), 4.19 (dd, *J* = 11.0, 5.0 Hz, 1H, H-6), 3.61 (t, *J* = 11.0 Hz, 1H, H-6), 3.44 (s, 1H, H-6a), 3.32 (d, *J* = 7.2 Hz, 2H, H-1'), 1.81 – 1.76 (S, 6H, H-4',5'). ^{13}C NMR (126 MHz, $CDCl_3$) δ 155.8 (C-3), 154.9 (C-4a), 154.2 (C-10a), 148.1 (C-9a), 141.7 (C-8a), 134.8 (C-3'), 131.8 (C-1), 121.9 (C-2'), 121.2 (C-3), 118.1 (C-7a), 112.2 (C-1a), 104.8 (C-7), 103.9 (C-4), 101.3 (C-2"), 93.8 (C-10), 78.6 (C-11a), 77.28, 77.03, 76.77, 66.5 (C-6), 40.2 (C-6a), 29.2 (1'), 25.8 (C-5'), 17.9 (C-4').

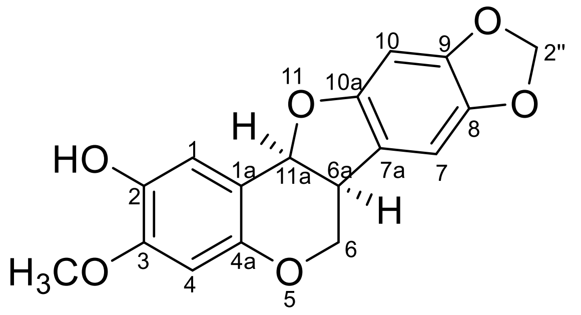

Compound 15 ((-)-2-hydroxypterocarpin) Data

Mp 223-225 °C (Lit.[8] 238-239 °C) $[\alpha]^{23}$ -107.4°(*c* 0.3 in $CHCl_3$) [Lit.[8] $[\alpha]^{23}$ -227.7° (*c* 0.8 in $CHCl_3$)], Its HRESITOFMS data afforded an [M + H] + ion at *m/z* 315.0869, implying a molecular formula of $C_{17}H_{14}O_6$ (calcd for $C_{17}H_{14}O_6$, *m/z* 315.0869). ^{1}H NMR (400 MHz, Acetone) δ 6.90 (s, 1H, H-7), 6.89 (s, 1H, H-1), 6.47 (s, 1H, H-10), 6.40 (s, 1H, H-4), 5.93 (d, *J* = 1.0 Hz, 1H, H-2"), 5.91 (d, *J* = 1.0 Hz, 1H, H-2"), 5.62 (s, OH), 5.47 (d, *J* = 6.9 Hz, 1H, H-11a), 4.27 – 4.23 (m, 1H, H-6), 3.82 (s, 3H, OCH_3), 3.65 – 3.58 (m, 1H, H-6), 3.71 – 3.47 (m, 1H, H-6a). ^{13}C NMR (101 MHz, Acetone) δ 154.4 (C-10a), 149.1 (C-4a), 147.9 (C-9), 141.6 (C-8), 143.3 (C-3), 141.3 (C-2), 118.6 (C-7a), 115.7 (C-1), 112.1 (C-1a), 104.9 (C-7), 101.2 (C-2"), 100.4 (C-4), 93.1 (C-10), 78.5 (C-11a), 66.3 (C-6), 55.3 (OCH_3).

Table S7. Experimental and Literature ^{13}C NMR Data of Compounds **19** and **20**

**Position**	**H δ 19 (CD_3OD)**	**(CD_3OD)**	**H δ 20 (CD_3OD)**	**CD_3OD**
	Expt.	Lit.[9]	Expt.	Lit.[9]
1	131.8	133.3	126.3	127.7
2	113.9	115.4	121.1	122.5
3	154.1	155.6	161.3	162.7
4	103.8	105.3	97.2	98.6
6	66.3	67.7	66.4	67.8
7	124.6	126.0	124.6	126.0
8	107.3	108.7	107.3	108.7
9	160.5	159.9	160.5	158.8
10	97.3	98.8	97.3	98.8

1a	112.9	114.4	112.1	113.6
4a	154.9	156.4	155.9	157.4
6a	39.6	41.1	39.5	40.9
7a	118.1	119.5	118.2	119.0
10a	158.4	162.0	158.4	162.0
11a	78.5	79.9	78.9	80.4
1'	30.1	31.6	29.3	30.8
2'	77.0	70.6	89.9	91.4
3'	69.2	78.4	71.2	72.5
4'	19.6	21.0	23.7	25.2
5'	24.5	26.0		
6'			23.9	25.4

Table S8. Experimental and Literature ^{13}C NMR Data of Compounds **2, 6, 7, 9** and **10**

P	H δ **2** ($CDCl_3$)		H δ **6** ($CDCl_3$)		H δ **7** ($CDCl_3$)		H δ **9** ($CDCl_3$)		H δ **10** ($CDCl_3$)	
	Expt.	Lit.[10]	Expt.	Lit.[10]	Expt.	Lit.[10]	Expt.	Lit.[10]	Expt.	Lit.[10]
1	122.8	122.9	-------	-------	-------	------	106.8	106.8	106.9	106.9
2	122.4	122.4	160.1	173.2	71.3	71.3	142.3	142.3	142.4	143.2
3	155.7	155.7	123.9	124.0	48.33	48.3	149.5	149.5	147.4	147.9
4	99.9	99.9	142.4	142.4	192.7	192.6	99.2	99.9	98.9	98.9
5	-------	-------	119.6	119.6	120.9	120.8	-------	-------	-------	-------
6	66.9	67.0	124.8	124.8	115.5	115.6	63.9	63.9	66.4	66.4
7	104.7	104.7	156.2	156.2	159.2	159.2	-------	-------	-------	-------
8	141.8	141.8	99.5	99.5	99.7	99.6	100.0	100.0	99.9	99.8
9	148.2	148.2	------	--------	-------	-------	158.3	158.3	158.6	158.6
10	93.8	93.8	------	--------	-------	--------	123.3	123.3	123.1	123.1
11	-------	--------	------	--------	-------	--------	121.0	121.0	121.0	121.0
12	-------	--------	------	151.7	-------	122.6	192.9	192.9	190.6	190.6-
1′	-------	--------	122.9	95.5	122.6	152.8	-------	--------	-------	--------
2′	-------	--------	151.6	148.8	152.7	95.4	-------	--------	-------	--------
3′	-------	--------	95.5	141.3	95.4	147.8	-------	--------	-------	--------
4′	-------	--------	148.7	110.3-	147.8	141.4	-------	--------	-------	--------
5′	-------	-------	141.1	--------	141.3	109.8	-------	--------	-------	-------
6′	-------	145.1	110.3	146.7	109.8	--------	-------	--------	-------	146.2
7′	-------	106.3	-------	106.4	-------	------	-------	146.4	-------	106.9
8′	-------	101.3	-------	101.5-	-------	146.0	-------	105.7	-------	101.2
2″	145.1	116.5	146.7	------	146.0	107.0	146.5	101.3	146.2	105.3
3″	106.2	153.5	106.4	116.2-	107.1	101.3	105.7	109.2	106.9	148.5
2‴	101.3	40.6	101.5 ------	--------	101.3	-------	101.3 109.2	149.6	101.2 105.3	72.1

1a<br>4a<br>6a<br>7a<br>8a<br>10a<br>11a<br>12a<br>OCH_3	116.5<br>153.5<br>40.6<br>117.9<br>-------<br>154.2<br>79.2<br>-------<br>-------	117.9<br>-------<br>154.2<br>79.2<br>--------<br>--------	116.2<br>------<br>------<br>157.8<br>-------<br>-------<br>-------<br>56.9	156.2-<br>--------<br>--------<br>--------<br>56.9	------<br>118.8<br>-------<br>-------<br>159.9<br>-------<br>-------<br>-------<br>56.5	118.8-<br>--------<br>--------<br>159.9<br>--------<br>--------<br>--------<br>56.5	149.6<br>75.9<br>160.3<br>-------<br>-------<br>114.6<br>68.3<br>-------	75.9<br>160.3<br>-------<br>-------<br>114.6<br>68.3<br>-------	148.5<br>72.1<br>159.8<br>-------<br>-------<br>116.1<br>45.3<br>-------	159.8<br>-------<br>-------<br>116.1<br>45.3<br>-------

Table S9. Experimental and Literature ^{13}C NMR Data of Compounds **14, 16, 17,** and **18**

**P**	**H** δ **14** ($CDCl_3$)	**H** δ **16** ($CDCl_3$)	**H** δ **17** ($CDCl_3$)	**H** δ **18** ($CDCl_3$)

	Expt.	Lit.[11]	Expt.	Lit.[12, 13]	Expt.	Lit.[10, 12]	Expt.	Lit.[10]
1	-------	-------	108.7	108.8	110.4	110.1	-------	------
2	66.5	66.5	142.9	142.9	157.9	157.4	154.7	154.7
3	39.4	39.4	151.1	151.2	167.4	166.8	121.1	121.1
4	78.3	76.5	101.0	101.1	100.9	100.6	176.6	176.6
5	132.3	132.2	-------	-------	-------	-------	119.0	119.0
6	109.7	109.8	63.8	63.9	66.3	65.9	126.0	126.0
7	160.2	160.2	-------	-------	-------	-------	157.3	157.2
8	103.6	103.7	113.2	113.2	112.9	112.9	99.8	99.8
9	156.7	157.2	168.1	168.0	149.5	149.0	-------	-------
10	112.6	112.4	105.3	105.3	104.9	104.4	-------	-------
11	-------	-------	130.1	130.2	130.0	129.4	-------	-------
12	-------	-------	191.1	191.1	188.9	188.3	-------	-------
1′	119.0	119.4	-------	--------	--------	----------	112.9	112.8
2′	122.1	122.0	-------	--------	-------	----------	153.0	153.0
3′	114.8	114.9	-------	--------	-------	----------	95.5	95.5
4′	154.5	156.6	31.1	31.1	31.3	30.9	148.5	148.4
5′	99.4	99.4	87.9	88.0	87.9	87.3	141.3	141.2
6′	156.9	154.4	144.0	142.9	143.1	142.1	111.3	113.3
7′	--------	--------	112.7	112.6	112.6	112.5	-------	-------
8′	--------	--------	17.1	17.1	17.1	16.7	-------	-------
2′′	76.8	78.4	-------	--------	-------	----------	147.4	147.4
3′′	127.6	127.6	-------	--------	-------	----------	107.1	107.0
2′′′	--------	--------	-------	--------	-------	----------	101.4	101.4
4′′	121.9 -------- 28.0	122.1	109.3	109.5	104.8	104.5	-------	-------

4a	27.8	--------	148.4	148.4	143.9	143.4	121.2	121.1
4′′′		--------	76.0	76.1	72.2	71.8	-------	--------
5′′′		26.9	157.7	157.7	147.4	147.0	-------	--------
8a			-------	--------	-------	----------	154.3	154.2
10a			-------	--------	-------	----------	-------	--------
11a			111.7	111.8	113.4	113.1	-------	--------
12a			67.6	67.6	44.6	44.1	-------	--------
OCH_3			55.9<br>56.4	55.9<br>56.4	55.9<br>56.3	55.9<br>56.4	56.9	56.9

Table S10. Crystal Data and Structure Refinement for Compound **11**

Identification code exp_80 CD2

Empirical formula	$C_{21}H_{20}O_5$
Formula weight	352.37
Temperature/K	149.99(10)
Crystal system	Monoclinic
Space group	$P2_1$
a/Å	4.49030(10)
b/Å	28.1187(4)
c/Å	13.8306(3)
α/°	90
β/°	91.272(2)
γ/°	90
Volume/$Å^3$	1745.84(6)
Z	4
$\rho_{calc}g/cm^3$	1.341
μ/mm^{-1}	0.095
F(000)	744.0
Crystal size/mm^3	0.41 × 0.22 × 0.09
Radiation	MoKα (λ = 0.71073)
2Θ range for data collection/°	4.132 to 61.38
Index ranges	-6 ≤ h ≤ 6, -40 ≤ k ≤ 40, -19 ≤ l ≤ 19
Reflections collected	53882
Independent reflections	10507 [R_{int} = 0.0428, R_{sigma} = 0.0359]
Data/restraints/parameters	10507/1/475
Goodness-of-fit on F^2	1.021
Final R indexes [I>=2σ (I)]	R_1 = 0.0398, wR_2 = 0.0935
Final R indexes [all data]	R_1 = 0.0519, wR_2 = 0.0992
Largest diff. peak/hole / e $Å^{-3}$	0.24/-0.17
Flack parameter	-0.2(3)

Table S11. Crystal Data and Structure Refinement for Compound **20**

Code; pyn1808SNQ

$C_{20}H_{20}O_5$
Mr = 340.38
Monoclinic, P21
Hall symbol: P 2yb
a = 10.9525 (1) Å
b = 6.5457 (1) Å
c = 11.3823 (1) Å
β = 98.6335 (8)°
V = 806.77 (2) Å3
Z = 2

F(000) = 360
Dx = 1.401 Mg m−3
Cu Kα radiation, λ = 1.54184 Å
Cell parameters from 14561 reflections
θ = 5–74°
µ = 0.83 mm−1
T = 150 K
Prism, colourless
0.31 × 0.12 × 0.10 mm

Data collection

Super Nova, Dual, Cu at zero, EosS2 diffractometer
Radiation source: micro-focus sealed X-ray tube,
Super Nova (Cu) X-ray Source
Mirror monochromator
ω scans

Absorption correction: multi-scan
Crys Alis PRO 1.171.39.46 (Rigaku Oxford Diffraction, 2018) Empirical absorption correction

using spherical harmonics, implemented in SCALE ABSPACK scaling algorithm.
Tmin = 0.70, Tmax = 0.92
19610 measured reflections
3089 independent reflections
3064 reflections with I > 2.0σ(I)
Rint = 0.031
θmax = 73.7°, θmin = 3.9°
h = −13→13
k = −7→8
l = −14→14

Refinement

Refinement on F2
Least-squares matrix: full
R[F2 > 2σ(F2)] = 0.035
wR(F2) = 0.094
S = 1.00
3089 reflections
233 parameters
1 restraint
Primary atom site location: structure-invariant direct
methods
Hydrogen site location: difference Fourier map

H atoms treated by a mixture of independent and constrained refinement
Method = Modified Sheldrick w = 1/[σ2(F2) + (0.08P)2 + 0.06P] ,
where P = (max(Fo 2,0) + 2Fc 2)/3
(Δ/σ)max = 0.0004
Δρmax = 0.22 e Å−3
Δρmin = −0.23 e Å−3
Absolute structure: Flack (1983), 1307 Friedel-pair

Absolute structure parameter: 0.19 (13)

Compounds	Melting Point (°C)		Optical rotation	
	Expt.	Lit.	Expt.	Lit.
**1**	--------	221.5 -233 [1]	$[\alpha]^{21}$ +12.9° (*c* 0.3 in $CHCl_3$)	---------
**2**	210	225 [10]	$[\alpha]^{25}$ -176° (*c* 0.3 in $CHCl_3$)	---------
**3**	69-71	--------	$[\alpha]^{21}$ +18.9° (*c* 0.3 in $CHCl_3$)	---------
**4**	--------	125-127 [3]	$[\alpha]^{25}$ -192° (*c* 0.05 in $CHCl_3$)	$[\alpha]_D$ -214° (*c* 0.01 in $CHCl_3$) [3]
**5**	150-152	170-171 [14]	$[\alpha]^{21}$ -43.2° (*c* 0.2 in $CHCl_3$)	$[\alpha]^{22}$ -51° (*c* 2 in $CHCl_3$) [14]
**6**	204-205	204-205 [10]	$[\alpha]^{26}$ -189.5° (*c* 0.01 in $CHCl_3$)	$[\alpha]_D$ 0° (Benzene or MeOH) [10]
**7**	174-175	180-181 [10]	$[\alpha]^{26}$ -232.2° (*c* 0.4 $CHCl_3$)	$[\alpha]_D$ 0° ($CHCl_3$ or Benzene) [10]
**8**	133-135	--------	$[\alpha]^{22}$ -11.2° (*c* 0.2 $CHCl_3$)	--------
**9**	155-156	189 [10]	$[\alpha]^{23}$ -173.2° (*c* 0.3 in $CHCl_3$)	--------
**10**	220	235 [10]	$[\alpha]^{23}$ +139.7° (*c* 0.2 in $CHCl_3$)	$[\alpha]^{23}$ +135° (*c* 1.3 in $CHCl_3$)[10]
**11**	149-152	146-147 [7]	$[\alpha]^{23}$ -339.5° (*c* 0.1 in $CHCl_3$)	$[\alpha]^{23}$ -261.9° (*c* 0.8 in $CHCl_3$) [7]
**12**	---------	115-116 [5]	$[\alpha]^{24}$ -102.2° (*c* 0.8 in $CHCl_3$)	$[\alpha]^{20}$ 0° ($CHCL_3$) [4]
**13**	172-174	93-95 [6]	$[\alpha]^{21}$ -127.2° (*c* 0.2 in $CHCl_3$)	$[\alpha]_D$ -273° (*c* 0.08 in $CHCl_3$) [1]
**14**	---------	154-155 [11]	$[\alpha]^{21}$ -234.6° (*c* 0.1 in $CHCl_3$)	$[\alpha]^{22}$ -291° (*c* 0.3 in $CHCl_3$) [11]
**15**	223-225	238-239 [8]	$[\alpha]^{23}$ -107.4°(*c* 0.3 in $CHCl_3$)	$[\alpha]^{23}$ -227.7° (*c* 0.8 in $CHCl_3$) [8]
**16**	159-162	162-164 [12]	$[\alpha]^{24}$ -236° (*c* 0.3 in $CHCl_3$)	$[\alpha]_D$ -199° ($CHCl_3$) [12]
**17**	152-154	87-90 [10, 12]	$[\alpha]^{20}$ -59.2° (*c* 0.5 in $CHCl_3$)	----------
**18**	237-239	239-240 [10]	$[\alpha]^{26}$ -152.6° (*c* 0.1 in $CHCl_3$)	----------
**19**	194-196	146-147 [9]	$[\alpha]^{21}$ -161.4° (*c* 0.1 in MeOH)	$[\alpha]^{23}$ -201.0° (*c* 0.8 in MeOH) [9]
**20**	209-213	215-216 [9]	$[\alpha]^{21}$ -208.5° (*c* 0.1 in MeOH)	$[\alpha]^{23}$ -194.5° (*c* 0.6 in MeOH) [9]

Table S12. Melting Point and Optical Rotation Literature and experimental Data of Compounds **1-20**

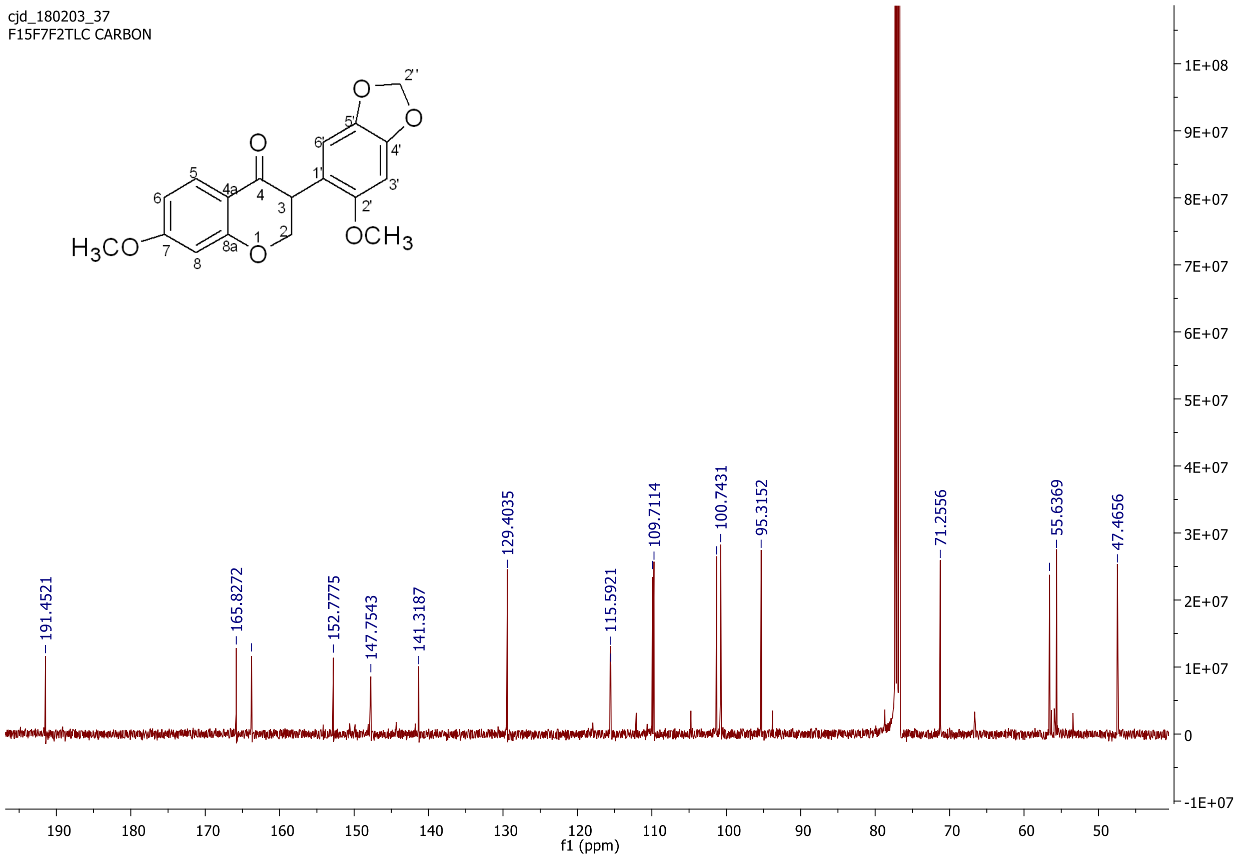

Figure S20. ^{13}CNMR Spectrum of Compound **8** (126 MHz, $CDCl_3$)

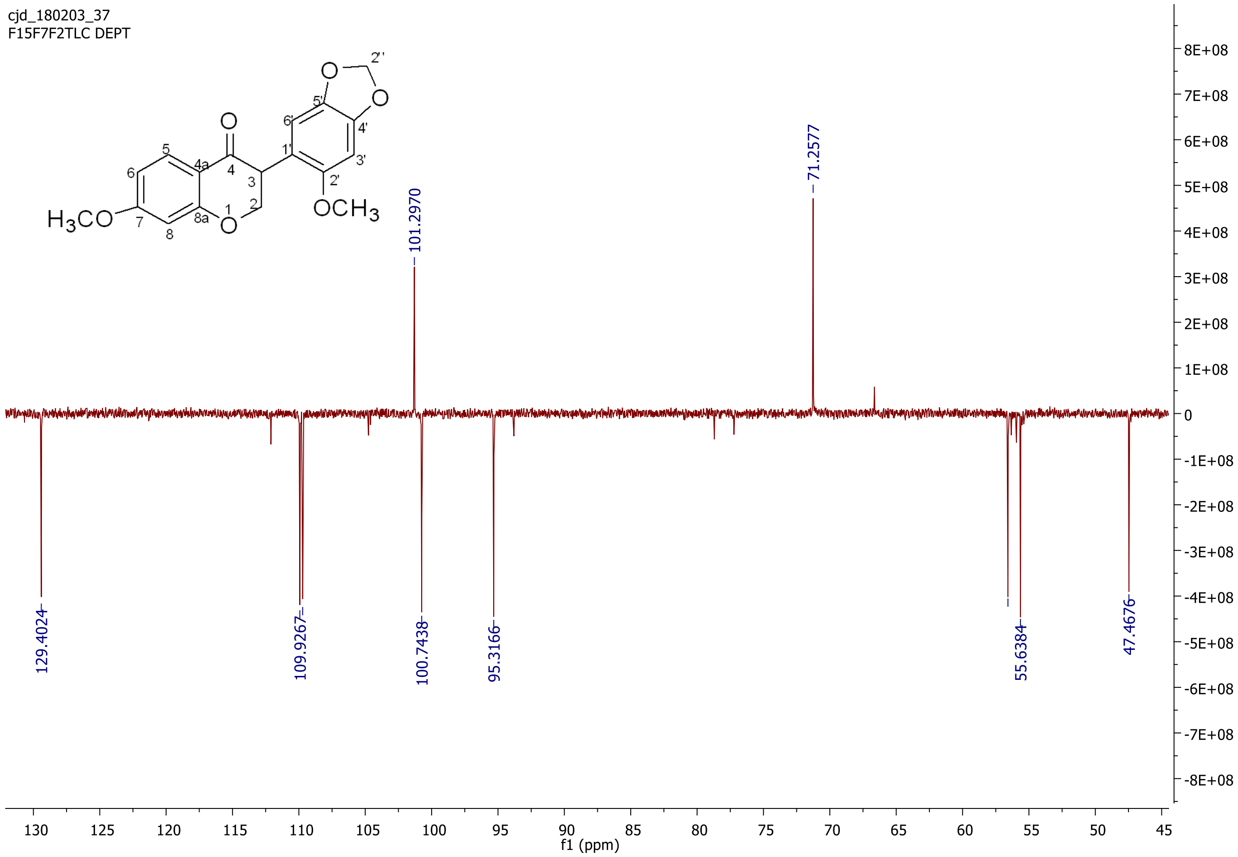

Figure S21. DEPT Spectrum of Compound **8** (500 MHz, $CDCl_3$)

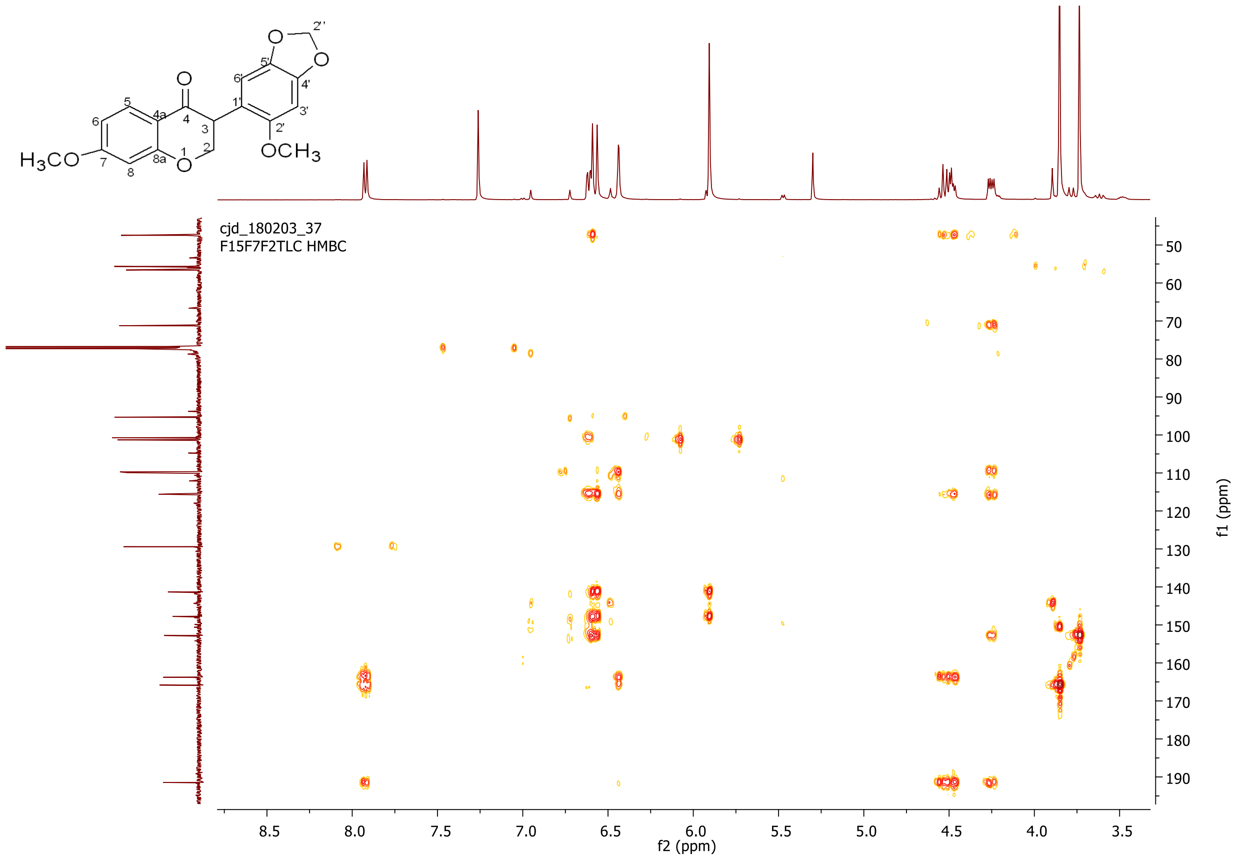

Figure S22. HSQC Spectrum of Compound **8** (500 MHz, $CDCl_3$)

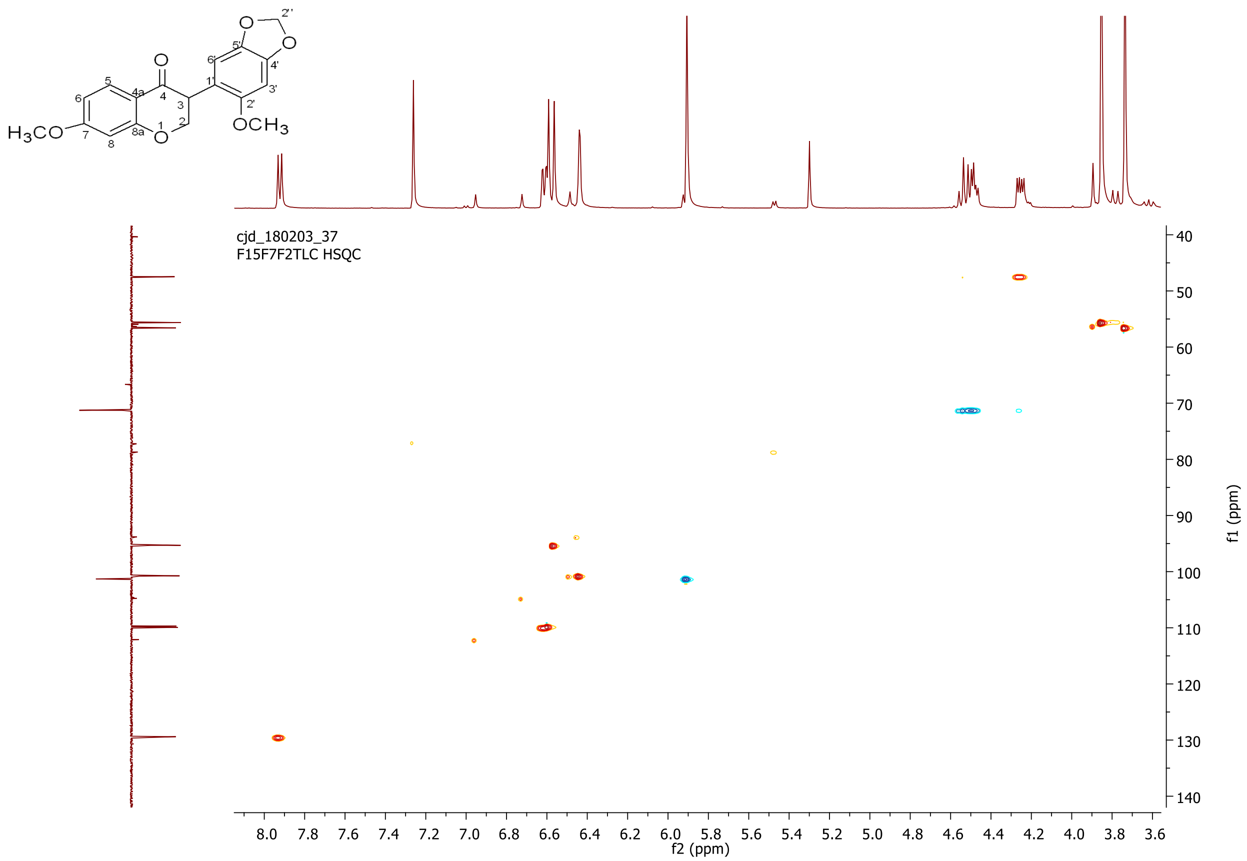

Figure S23. HSQC Spectrum of Compound **8** (500 MHz, $CDCl_3$)

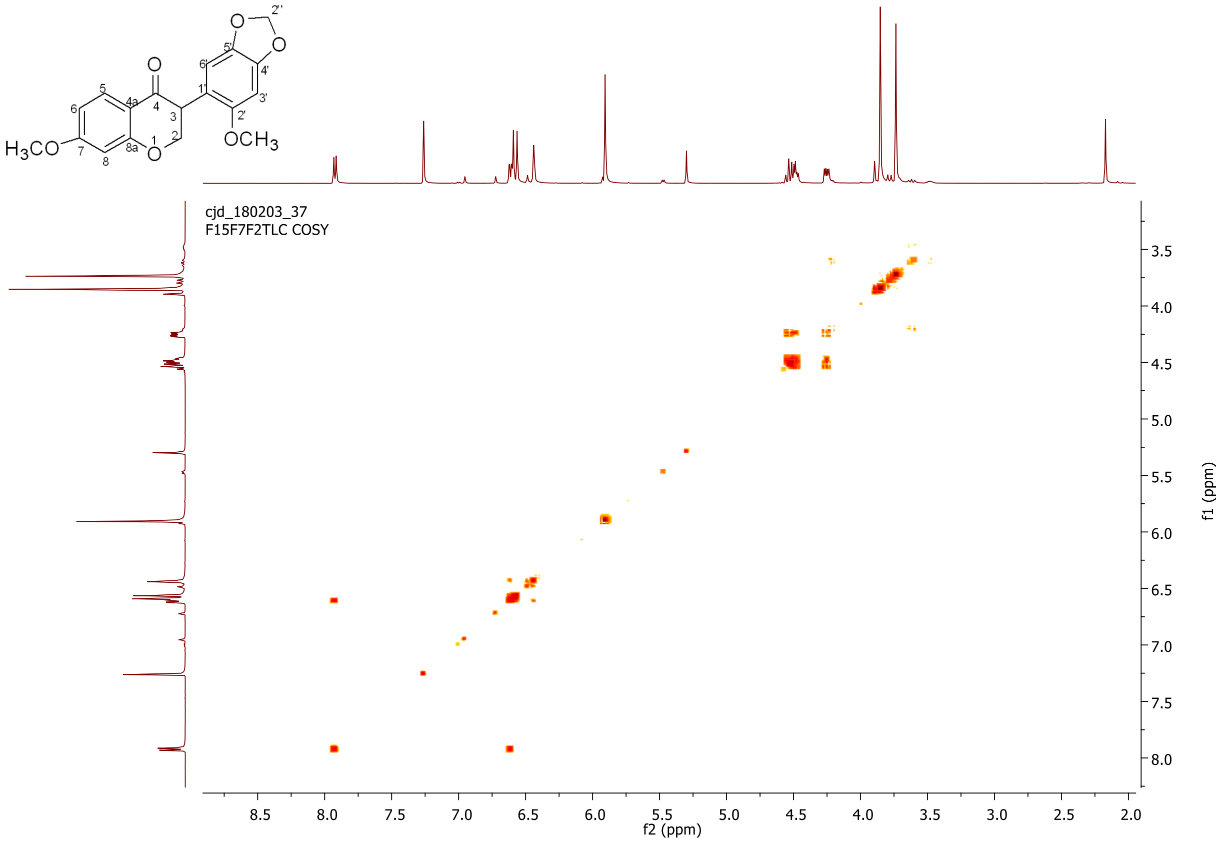

Figure S24. COSY Spectrum of Compound **8** (500 MHz, $CDCl_3$)

Elemental Composition Report

Page 1

Single Mass Analysis
Tolerance = 5.0 PPM / DBE: min = -1.5, max = 120.0
Element prediction: Off
Number of isotope peaks used for i-FIT = 3

Monoisotopic Mass, Even Electron Ions
746 formula(e) evaluated with 4 results within limits (up to 15 closest results for each mass)
Elements Used:
C: 9-44 H: 0-75 N: 0-10 O: 0-12 Na: 0-1
F15F7F2TLC
SP_CDawurung F15F7F2TLC 88 (2.108) Cm (81:89)

1: TOF MS ES+
5.54e+003

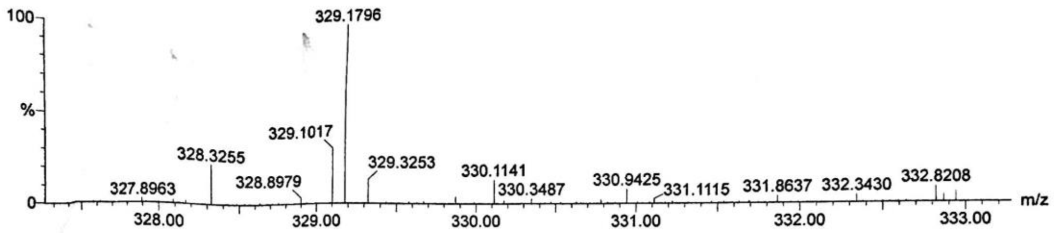

Mass	Calc. Mass	mDa	PPM	DBE	i-FIT	i-FIT (Norm)	Formula
Minimum:				-1.5			
Maximum:		55.0	5.0	120.0			
329.1017	329.1014	0.3	0.9	12.5	278.6	1.4	C17 H14 N4 O2 Na
	329.1012	0.5	1.5	16.5	279.1	1.9	C15 H9 N10
	329.1025	-0.8	-2.4	10.5	278.0	0.8	C18 H17 O6 ✓
	329.1001	1.6	4.9	7.5	279.1	1.8	C16 H18 O6 Na

MS for compound 8

References

1. Brink, A.J., Rall, G.J.H., Engelbrecht, J., 1974. Structures of some minor pterocarpans of *Neorautanenia edulis.* Phytochemistry 8, 1581-1585. https://doi.org/10.1016/0031-9422 (74)80332-8.
2. Fraga, B.M., Gonzalez-Coloma A., Alegre-Gomez, S., Lopez-Rodriguez, M., Amador, L. J., Daiz, C. E., 2017. Bioactive constituents from transformed root cultures of *Nepeta teydea*. Phytochemistry. 133:59-69. https://doi.org/10.1016/j.phytochem.2016.10.008.
3. Breytenbach, J.C., Rall, G.J.H., 1980. Structure and synthesis of isoflavonoid analogues from *Neorautanenia amboensis* Schinz. J. Chem. Soc, Perkin Trans. 0, 1804-1809. https://doi.org/10.1039/P19800001804.
4. Crombie, L., Whiting, D. A., 1963. The extractives of *Neorautanenia pseudopachyrrhiza* the isolation and structure of a new rotenoid and two isoflavanones. J. Chem. Soc. 1963, 1569-1579.
5. Mitscher, L.A., Okwute, S.K., Gollapudi, S.R., Drake, S., Avona, E., 1988. Antimicrobial pterocarpans of Nigerian *Erythrina mildbraedii*. Phytochemistry. 11, 3449-3452. https://doi.org/10.1016/0031-9422(88)80746-5.
6. Rall, G.J.H., Engelbreght, J.P., Brink, A.J., 1971. The chemistry of *Neorautanenia edulis* G.A, Sm. The constitution of (-)-2-isopentenyl-3-hydoxy-8-9 methylenedioxypterocarpan, a new pterocarpan from the root bark. J. South Afri. Chem. Inst. 26, 56-60.
7. Rall, G.J.H., Engelbreght, J.P., Brink, A.J., 1970. The isolation, structure and absolute configuration of (-)-2-hydroxypterocarpin from *Neorautenenia edulis*. Tetrahedron**.** 26, 507-5012.
8. Sakurai, Y., Sakurai, N., Masahiko, T., Nakanishi, Y., Bastow, K.F., Wang, X., Cragg, G.M., Lee, K.H., 2006. Rautandiols A and B, pterocarpans and cytotoxic constituents from *Neorautanenia mitis* J. Nat. Prod., 69, 397-399. https://doi.org/10.1021/np058070c.
9. Puyvelde, L.V., Norbert, D., Kimpe, N.D., Jean-Pierre, Mudaheranwa, J.P., Gasiga, A., Schamp, N., Jean-Paul, Declercq, J.P., Meerssche, M.V., 1987. Isolation and structure elucidation of potentially insecticidal and acaricidal isoflovone- type compounds from *Neorautanenia mitis*. J. Nat. Prod. 50, 349-56. https://doi.org/10.1021/np50051a002.
10. Nkengfack, A.E., Vardamides, J.C., Z.; Tanee Fomum, Z.T., Meyer, M., 1995. Prenylated Isoflavanone from *Erythrina eriotricha*. Phytochemistry. 6, 1803-1808. https://doi.org/10.1016/0031-9422(95)00037-8.
11. Oberholzer, M.E., Rall, G.J.H., Roux, D.G., 1974. The concurrence of 12a-hydroxy- and 12a-O-methylrotenoids. Isolation of the first natural 12a-O-methylrotenoids. Tetrahedron Letters. 25, 2211-2214.
12. Phrutivorapongkul, A., Lipipun, V., Ruangrungsi, N., Watanabe, T., Ishikawa, T., 2002. Studies of the constituents of seeds of *Pachyrrhizus erosus* and their anti-herpes simplex virus (HSV) activities. Chem. Pharm. Bull. 50, 534-537. https://doi.org/10.1248/cpb.50.534.
13. Chaturvedula, V.S.P., Prakash, I., 2012. Isolation of Stigmasterol and β-Sitosterol from the dichloromethane extract of *Rubus suavissimus*. ICPJ. 9, 239-242. https://doi.org/10.3329/icpj.v1i9.11613.

14. Van Duuren, B.L., 1961. Chemistry of edulin, Neorautone, and related compounds from *Neorautanenia edulis* C.A. Sm. J. Org. Chem. 12, 5013-5020. https://doi.org/10.1021/jo01070a057.
